# Identification of candidate selenium-associated transcriptomic biomarkers in chronic obstructive pulmonary disease: an integrated bioinformatics and expression validation study

**DOI:** 10.3389/fmed.2026.1909444

**Published:** 2026-09-30

**Authors:** Yang Li, Zhuxiu Jiang, Zhibo Xu, Ying Chen, Meihua Wang, Yuanting Wu

**Affiliations:** Department of Respiratory Medicine, Hangzhou Xixi Hospital Affiliated to Zhejiang Chinese Medical University, Hangzhou, China

**Keywords:** chronic obstructive pulmonary disease, nomogram, ribosome, selenium metabolism and selenoprotein, WGCNA

## Abstract

**Introduction:**

Chronic obstructive pulmonary disease (COPD) is marked by long-lasting airflow obstruction accompanied by persistent inflammation and disturbed redox balance. Although oxidative stress is well established in COPD, it remains unclear whether PBMC transcriptional patterns that co-vary with a curated selenium-metabolism and selenoprotein signature can identify candidate COPD markers. We therefore evaluated such patterns as an exploratory transcriptomic prioritization strategy rather than as evidence of selenium-specific regulation.

**Methods:**

COPD-related transcriptomic datasets were downloaded from the GEO database, and differentially expressed genes (DEGs) were determined using conventional differential expression analysis. Selenium metabolism and selenoprotein (SMASP) genes were collected from the MSigDB. WGCNA was then used to identify a module closely associated with COPD status and SMASP scores, and candidate genes were defined by intersecting module genes with DEGs. XGBoost and SVM-RFE, together with cross-validation, were applied to select biomarkers. A nomogram was subsequently generated to assess their diagnostic utility. Gene set enrichment analysis (GSEA) and RT-qPCR validation were finally conducted to examine the potential involvement of these biomarkers in COPD.

**Results:**

We detected 235 DEGs and 40 selenoprotein-related genes, from which 17 overlapping candidate genes were obtained. ERBB4 and ZKSCAN3 were ultimately retained as biomarkers and were significantly decreased in COPD samples. The nomogram based on these two genes showed favorable predictive ability. GSEA indicated that both biomarkers were associated with ribosome and olfactory transduction pathways. ERBB4 was also linked to neuroactive ligand-receptor interaction, whereas ZKSCAN3 was related to extracellular matrix (ECM)-receptor interaction. RT-qPCR in a small exploratory cohort (*n* = 5 per group) showed directionally consistent downregulation of ERBB4 and ZKSCAN3 in COPD samples, supporting the bioinformatics findings. This exploratory analysis was cross-sectional and does not establish causality, selenium deficiency, or clinical utility. The findings are hypothesis-generating and require prospective validation.

**Conclusion:**

ERBB4 and ZKSCAN3 were prioritized as exploratory candidate expression markers in the analyzed COPD datasets. Their diagnostic value, selenium specificity, and biological functions require confirmation in larger independent cohorts and functional studies.

## 1 Introduction

COPD is a common progressive inflammatory airway disease characterized by persistent, incompletely reversible airflow limitation. Pathological injury mainly involves the small airways, lung parenchyma, and pulmonary vasculature, while typical clinical manifestations include chronic cough, sputum production, and exertional dyspnea that gradually worsens over time (1–3). Its global burden is still increasing: prevalent cases are estimated to rise from 213 million in 2021 to 592 million by 2050. Morbidity and mortality are particularly severe in regions with a low socio-demographic index (SDI), and COPD has become the third leading cause of death worldwide (4). The development of COPD reflects a prolonged interaction between genetic predisposition and environmental exposures, especially cigarette smoke, biomass fuel, and ambient air pollution. Chronic inflammation, oxidative injury, and protease-antiprotease disequilibrium constitute the major pathological features of the disease (1, 5). Importantly, COPD is not a uniform disorder. Patients differ substantially in clinical phenotypes, imaging patterns, and molecular endotypes, and the relative contribution of classical pathogenic pathways varies across subgroups that remain insufficiently defined (6, 7). Existing treatment strategies also have clear limitations. Although bronchodilators and inhaled corticosteroids are widely used to alleviate symptoms, they cannot repair established structural lung damage or fully prevent disease progression (6, 8). In routine primary care, inadequate guideline implementation, incorrect inhaler use, and limited availability of pulmonary rehabilitation further compromise disease control (8). Biologic agents designed for specific inflammatory endotypes are promising but still face difficulties related to patient selection, efficacy, and safety (6, 9). A clearer understanding of molecular targets and signaling pathways involved in COPD initiation and progression is therefore necessary to improve stratified therapy and prognostic management.

Selenium (Se) is an indispensable trace element incorporated as selenocysteine into at least 25 selenoproteins, forming a selenium-dependent antioxidant network that is functionally distinct from other antioxidant systems. This network operates through coordinated rather than isolated activities (10–12). Through this network, cells maintain redox homeostasis, immune protection, and endocrine regulation. Representative functions include ROS removal by GPX1, suppression of ferroptosis through GPX4, selenium transport mediated by selenoprotein P, and modulation of NF-*κ*B signaling (11, 13, 14). Unlike individual antioxidant enzymes (e.g., SOD or CAT), selenoproteins are uniquely dependent on dietary selenium availability and function as an integrated system for ROS elimination, lipid peroxide reduction, and inflammatory regulation. We therefore prioritized selenium metabolism and selenoprotein genes as a coherent functional module in COPD transcriptomic analysis, rather than analyzing general oxidative stress genes in isolation. Clinical observations have shown that selenium deficiency is common among individuals with COPD. Data from the NHANES indicate an inverse association between dietary selenium intake and COPD risk, with an odds ratio of 0.80 among participants in the highest intake category (9). Selenium status is also positively related to key pulmonary function indices, including forced expiratory volume in one second (FEV_1_) and forced vital capacity (FVC) (15). At the mechanistic level, selenium insufficiency can reduce GPX1 activity and weaken ROS clearance, thereby promoting epithelial injury and sustained inflammation in the lung. It may also compromise immune cell activity and increase susceptibility to respiratory infection, which is a major cause of acute COPD exacerbation (9, 11). Therapeutic studies further suggest that selenium supplementation may be beneficial in some COPD settings. Intravenous selenium may shorten the duration of mechanical ventilation in severe COPD, while combined selenomethionine supplementation can increase serum selenium by 58%, improve skeletal muscle strength, and reduce muscle weakness from 30% to 10.7% (11). Taken together, these findings indicate that selenoproteins may participate broadly in COPD development and progression. Although available evidence supports a close relationship between selenium/selenoprotein disturbance and COPD-related pathology, nutritional impairment, and adverse outcomes, the expression landscape and regulatory network of selenoproteins in COPD samples remain incompletely described. This study therefore sought to systematically profile selenoprotein-related expression patterns and explore their regulatory mechanisms in COPD, with the aim of informing selenium-based nutritional intervention and selenoprotein-targeted adjunctive therapy.

Here, transcriptomic data from the GEO database were combined with curated gene sets representing selenium metabolism and selenoprotein families. Differential expression analysis was integrated with two machine learning approaches, SVM-RFE and XGBoost, to identify selenium-related diagnostic biomarkers for COPD. The selected biomarkers were then evaluated using a nomogram, GeneMANIA network analysis, GSEA, and regulatory network visualization. In addition, RT-qPCR was used to verify their expression patterns and strengthen the reliability of the bioinformatics findings.

## 2 Materials and methods

### 2.1 Data collection

Two transcriptomic datasets from the GEO database were used to explore the molecular mechanisms associated with COPD. GSE248493, generated on the GPL18573 platform, served as the training dataset and contained 25 peripheral blood mononuclear cell (PBMC) samples from COPD patients and 12 samples from healthy controls. From the GSE248493 dataset, one COPD sample (GSM5908762) and one control sample (GSM5908752) were excluded because the raw data files were incomplete and could not be processed by the RMA normalization workflow. No other sample exclusion criteria were applied. GSE94916, generated on the GPL20844 platform, was used for validation and included 6 COPD PBMC samples and 6 control PBMC samples. Clinical information including age, sex, smoking status, GOLD stage, lung function parameters (FEV1, FVC, FEV1/FVC), medication use, comorbidities, and acute exacerbation status was not available in the public dataset. We were therefore unable to adjust for these variables. Forty-seven selenium metabolism and selenoprotein (SMASP) genes were collected from the WikiPathways term “WP_SELENIUM_METABOLISM_AND_SELENOPROTEINS” (16) within the C2 subset (Curated Canonical Pathways) of the MSigDB (Supplementary Table S1).

### 2.2 Screening of differentially expressed genes (DEGs)

For GSE248493, DEGs between COPD and control samples were screened using the “DESeq2” package(v1.42.0) (17), with thresholds of |log2 fold change (FC)| >1 and *P* < 0.05. Volcano plots were drawn with the “ggplot2” package(v3.5.2) (18), and heatmaps were produced using the “ComplexHeatmap” package(v2.21.1) (19). Genes were ranked according to the absolute value of log2 FC, and the top 10 upregulated and top 10 downregulated genes were displayed. DESeq2's internal filtering procedure excluded genes with very low expression (baseMean below a detection threshold) from multiple-testing correction, resulting in NA adjusted *P*-values. This primarily reflects low expression levels in PBMC samples (median baseMean = 5.5), which is biologically plausible for this tissue type. Differential expression was determined using |log_2_FC| >1 and nominal *P* < 0.05, with subsequent multi-step validation (WGCNA, machine learning, independent datasets, RT-qPCR) used to reduce false positives.

### 2.3 Screening of candidate genes

The enrichment score of SMASP genes in each sample was calculated by single-sample gene set enrichment analysis (ssGSEA) with the “GSVA” package(v1.50.0) (20). Score distributions in the COPD and control groups were plotted using “ggpubr” (v0.6.0), and differences were evaluated by the Wilcoxon rank-sum test (*P* < 0.05). Weighted gene co-expression network analysis was then performed on GSE248493 using the “WGCNA” package (v1.72.5). The pickSoftThreshold function was applied to determine a soft-thresholding power *β* that satisfied scale-free topology (coefficient of determination (R^2^) > 0.9). Modules were detected through dynamic tree cutting (minimum module size = 200, deepSplit = 2, mergeCutHeight = 0.25). Pearson correlation analysis was used to relate module eigengenes to SMASP scores, and modules with |correlation coefficient (r)| > 0.3 and *P* < 0.01 were considered significant. Within the key modules, genes with a gene significance (GS) >0.5 and a module membership (MM) >0.8 were identified; these genes were defined as genes co-expressed with the SMASP-derived score. This designation indicates the calculation of co-expression and does not imply that these genes are direct components of selenoproteins or selenium metabolism. Candidate genes were obtained by intersecting these key genes with DEGs and were illustrated using the “ggvenn” package(v0.1.10) (21).

### 2.4 Functional enrichment analysis of candidate genes

Functional annotation of candidate genes was carried out using the “clusterProfiler” package(v4.8.3) (22). GO and KEGG enrichment analyses were corrected for multiple comparisons using the Benjamini-Hochberg (BH) method. Results with a *P* adj < 0.05 were considered statistically significant; results with a nominal *P* < 0.05 but a *P* adj ≥ 0.05 were reported as exploratory findings and should be interpreted with caution. For GO enrichment, the 10 most significant terms in biological process (BP), cellular component (CC), and molecular function (MF) were selected according to *P*-value ranking. For KEGG analysis, the top 10 pathways were chosen using the same ranking strategy. The PPI network was generated through the STRING database, with a minimum interaction score >0.15.

### 2.5 Screening for biomarkers

Candidate genes were further prioritized using two machine learning approaches, SVM-RFE and XGBoost. Given the limited sample size, these algorithms were applied as exploratory feature-ranking approaches rather than as fully validated predictive models. SVM-RFE was implemented using the “caret” package(v6.0.94) (23) with internal 5-fold cross-validation for feature ranking. The “xgboost” package (version v2.0.3.1) (24) was used to assess the relative importance of features within the development dataset. Due to the lack of an independent external validation set, it was not possible to evaluate the model's performance (Supplementary Materials).

### 2.6 Diagnostic model development

A logistic regression-based nomogram for individualized COPD prediction was established using the “rms” package(v6.5.0) (25). Clinical usefulness was examined by DCA with the “ggDCA” package (v1.2). Calibration performance was assessed using calibration curves generated by the “regplot” package (v1.1, https://CRAN.R-project.org/package=regplot).

### 2.7 GeneMANIA network analysis

The identified biomarkers were submitted to the GeneMANIA online database (https://genemania.org), with Homo sapiens specified as the species. A gene interaction network was built from co-expression, physical interaction, pathway co-occurrence, and co-localization information to characterize possible regulatory relationships and functional modules involving the key genes.

### 2.8 GSEA

GSEA was used in GSE248493 to explore biological processes and potential functions related to the biomarkers. Spearman correlations between each biomarker and all remaining genes were calculated with the “corr.test” function in the “psych” package (v2.4.3). Genes were sorted in descending order according to correlation strength. Enrichment analysis was conducted using the “clusterProfiler” package, applying |normalized enrichment score (NES)| >1 and *P* < 0.05 as criteria. The most recent CP:KEGG gene set from MSigDB was downloaded through the “msigdbr” package (v7.5.1) and used as the reference gene set.

### 2.9 Regulatory network construction

The ENCORI database was used to predict miRNAs targeting the biomarkers. The resulting miRNA-messenger RNA (mRNA) interaction pairs were then imported into Cytoscape software (v3.8.2) for network construction and visualization, allowing potential post-transcriptional regulatory mechanisms to be examined.

### 2.10 RT-qPCR

RT-qPCR was conducted to verify biomarker expression in COPD and control samples. For experimental validation, peripheral blood samples were collected from five COPD patients and five control individuals. Clinical information, including demographic characteristics and available disease-related parameters, was collected and summarized in (Supplementary Table S8). COPD diagnosis was based on clinical assessment according to established criteria. Ten frozen tissue samples were obtained from Hangzhou Xixi Hospital. Written informed consent was obtained before sample collection, and the study was approved by the Hangzhou Xixi Hospital Ethics Review Committee (Approval No: HXMIRB-2026-047).

Total RNA was isolated from the 10 samples with TRIzol reagent (Novazene, China) following the manufacturer's protocol. RNA concentration and purity were assessed using the NanoPhotometer N50 (Implen, Germany). Reverse transcription was subsequently performed with the HP All-in-one qRT Master Mix II RT203-Ver.1 kit (Yungeng, China). RT-qPCR was carried out on the CFX Connect Real-Time PCR System (Bio-Rad, USA), and relative gene expression was determined by the 2−*ΔΔ*Ct method. The procedures followed those described previously (26). Primer sequences used for polymerase chain reaction amplification are listed in (Supplementary Table S2).

### 2.11 Statistical analysis

Statistical analyses were completed in R software (v4.3.3). Differences between groups were tested using the Wilcoxon rank-sum test or the t-test. *P < 0.05, **P < 0.01, ***P < 0.001, ****P < 0.0001, ns, not significant.

## 3 Results

### 3.1 Identification of candidate genes in COPD

Differential expression analysis identified 235 DEGs, comprising 42 upregulated and 193 downregulated genes. Because the PBMC transcriptome has limited expression breadth, adjusted *P*-values were unavailable for low-expression genes. The DEGs presented were identified by nominal *P* < 0.05. (Figures 1A,B; Supplementary Table S3). SMASP scores were significantly reduced in COPD samples (*P* < 0.05) (Figure 1C). Following hierarchical clustering-based outlier removal, *β* = 4 was chosen as the soft threshold (Figures 1D,E). Dynamic tree cutting detected eight modules after excluding the gray module (Figure 1F). The turquoise module, containing 4,288 genes, showed a significant association with SMASP scores (*r* = 0.50, *P* = 0.002) and was therefore selected as the key module. (Figure 1G). Genes within this module also displayed strong positive correlations between module membership and SMASP scores (|r| > 0.3, *P* < 0.001). By applying the selection criteria of GS >0.5 and MM >0.8, a total of 40 genes co-expressed with the SMASP-derived score were identified (Figure 1H; Supplementary Table S4). Intersecting these genes with the 235 DEGs produced 17 candidate genes: ASXL3, AXDND1, COL1A2, CPS1, CTNNA2, DNAH5, ERBB4, KCTD8, MEIS2, NANOG, NCAM2, PTCHD1, RBMS3, SEMA3E, SERTAD4, ZKSCAN3, and ZNF536 (Figure 1I).

**Figure 1:**
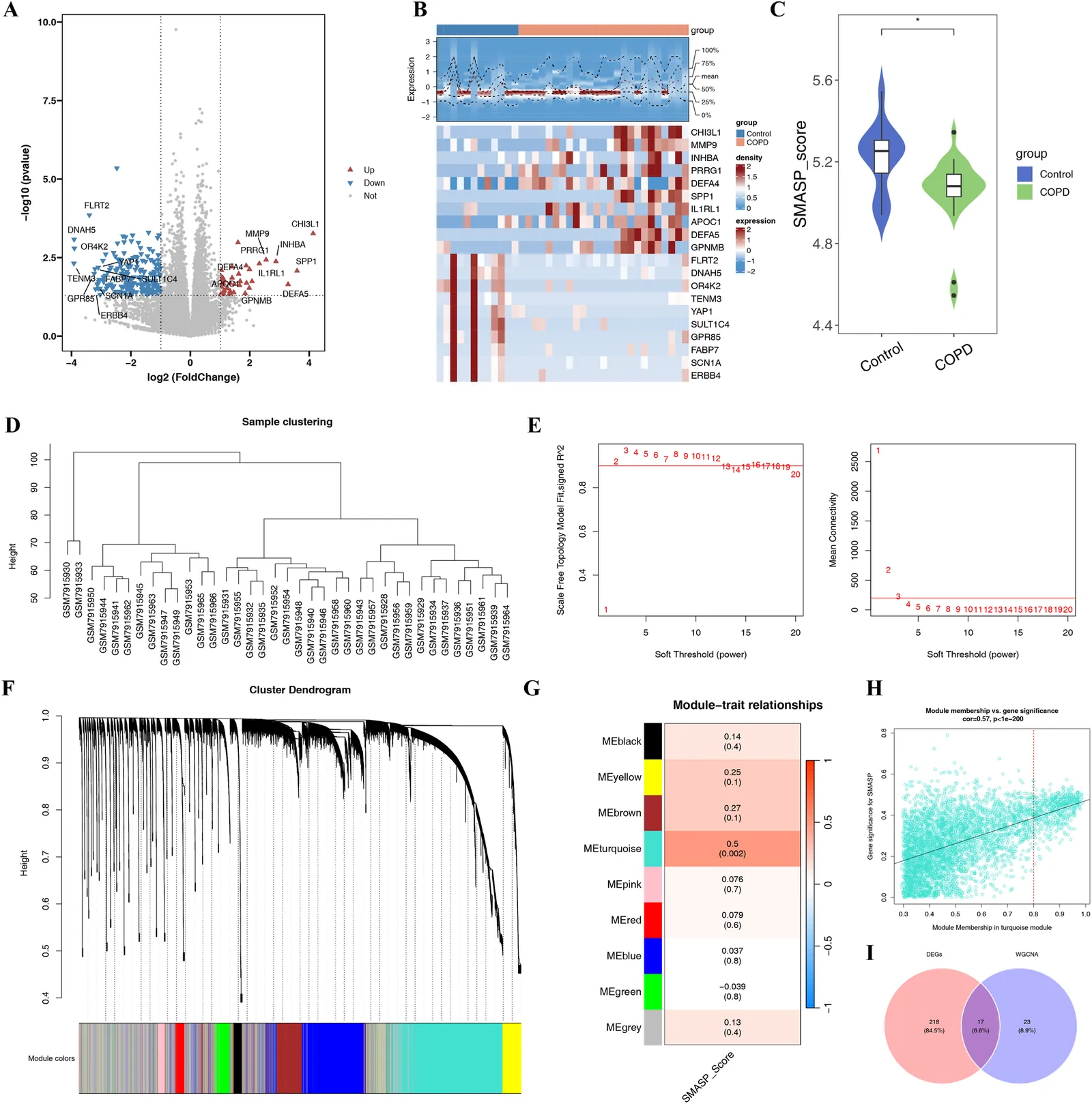
Identification of selenium metabolism and selenoprotein-related candidate genes in COPD. **(A)** Volcano plot showing differentially expressed genes (DEGs) between COPD and control samples in GSE248493. Red and blue dots indicate significantly upregulated and downregulated genes, respectively. **(B)** Heatmap displaying the top 10 upregulated and top 10 downregulated DEGs ranked by absolute log2 fold change. **(C)** Comparison of selenium metabolism and selenoprotein (SMASP) scores between COPD and control samples. **(D,E)** Determination of the soft-thresholding power in weighted gene co-expression network analysis (WGCNA), with *β* = 4 selected for network construction. **(F)** Gene co-expression modules identified by dynamic tree cutting. **(G)** Correlation heatmap between module eigengenes and clinical traits or SMASP scores. **(H)** Scatter plot showing the relationship between gene significance and module membership in the key module. **(I)** Venn diagram showing the intersection between DEGs and selenium metabolism and selenoprotein-related key genes, yielding 17 candidate genes. COPD, chronic obstructive pulmonary disease; DEG, differentially expressed gene; SMASP, selenium metabolism and selenoprotein; WGCNA, weighted gene co-expression network analysis.

### 3.2 Functional enrichment analysis of candidate genes

GO analysis revealed 169 significantly enriched terms, including 134 BP terms, 13 CC terms, and 22 MF terms (*P* < 0.05) (Figure 2A; Supplementary Table S5). Representative BP terms included neural crest cell migration, mesenchymal cell migration, and stem cell development. Representative CC terms included presynaptic membrane, banded collagen fibril, and complex of collagen trimers, whereas MF enrichment involved DNA-binding transcription repressor activity, peroxisome proliferator-activated receptor (PPAR) binding, and platelet-derived growth factor binding. KEGG analysis identified 7 enriched pathways (*P* < 0.05) (Figure 2B; Supplementary Table S6), including proteoglycans in cancer, phosphatidylinositol 3-kinase (PI3K)-protein kinase B (Akt) signaling pathway, nitrogen metabolism, and alanine, aspartate, and glutamate metabolism. Among the 7 KEGG pathways, only “Proteoglycans in cancer” reached statistical significance after correction (*P* adj < 0.05). The remaining pathways are reported as exploratory nominal findings and should be interpreted with caution. In the PPI network, 11 of the 17 candidate genes formed 16 interactions under a STRING interaction score >0.15 (Figure 2C).

**Figure 2:**
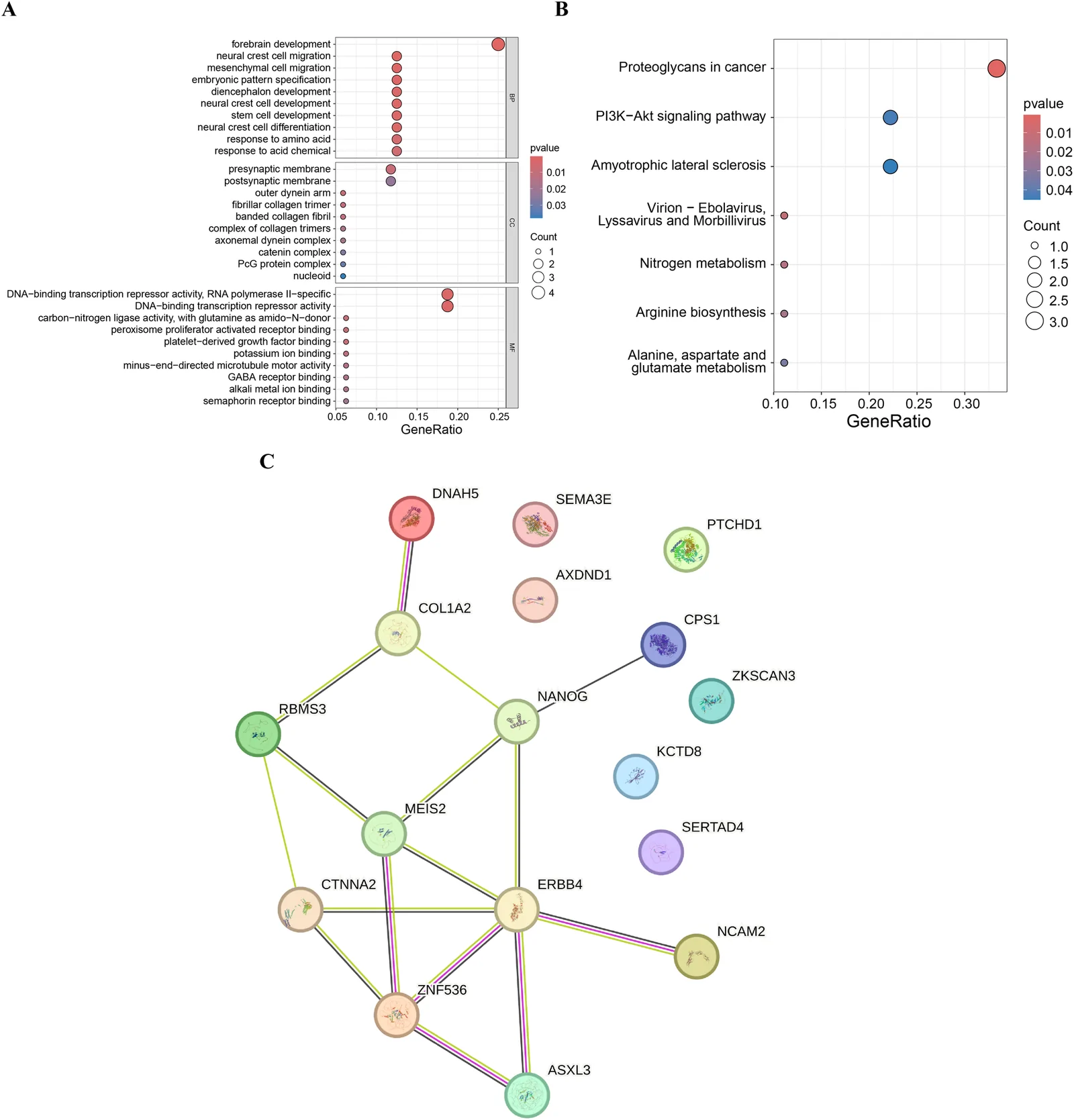
Functional enrichment and protein-protein interaction analyses of candidate genes. **(A)** Gene Ontology (GO) enrichment analysis of the 17 candidate genes, including biological process, cellular component, and molecular function categories. **(B)** Kyoto Encyclopedia of Genes and Genomes (KEGG) pathway enrichment analysis of the candidate genes. **(C)** Protein-protein interaction (PPI) network of candidate genes constructed using the STRING database with a minimum interaction score >0.15. GO, Gene Ontology; KEGG, Kyoto Encyclopedia of Genes and Genomes; PPI, protein-protein interaction.

### 3.3 Screening for biomarkers

SVM-RFE selected 13 genes (Figure 3A), while the XGBoost model assigned positive importance values to 10 genes (Figure 3B). Intersecting the outputs of these two machine learning algorithms yielded seven key genes: ERBB4, KCTD8, NCAM2, PTCHD1, SERTAD4, ZKSCAN3, and ZNF536 (Figure 3C). Among them, ERBB4 and ZKSCAN3 were the only genes that were consistently and significantly downregulated in both the training and validation datasets (*P* < 0.05) (Figures 3D,E). Given the limited sample size and the potential for instability in feature selection, these findings should be regarded as a basis for generating hypotheses; ERBB4 and ZKSCAN3 were retained as exploratory candidate markers because they satisfied the prespecified multistep screening rule; their inclusion does not constitute independent diagnostic validation.

**Figure 3:**
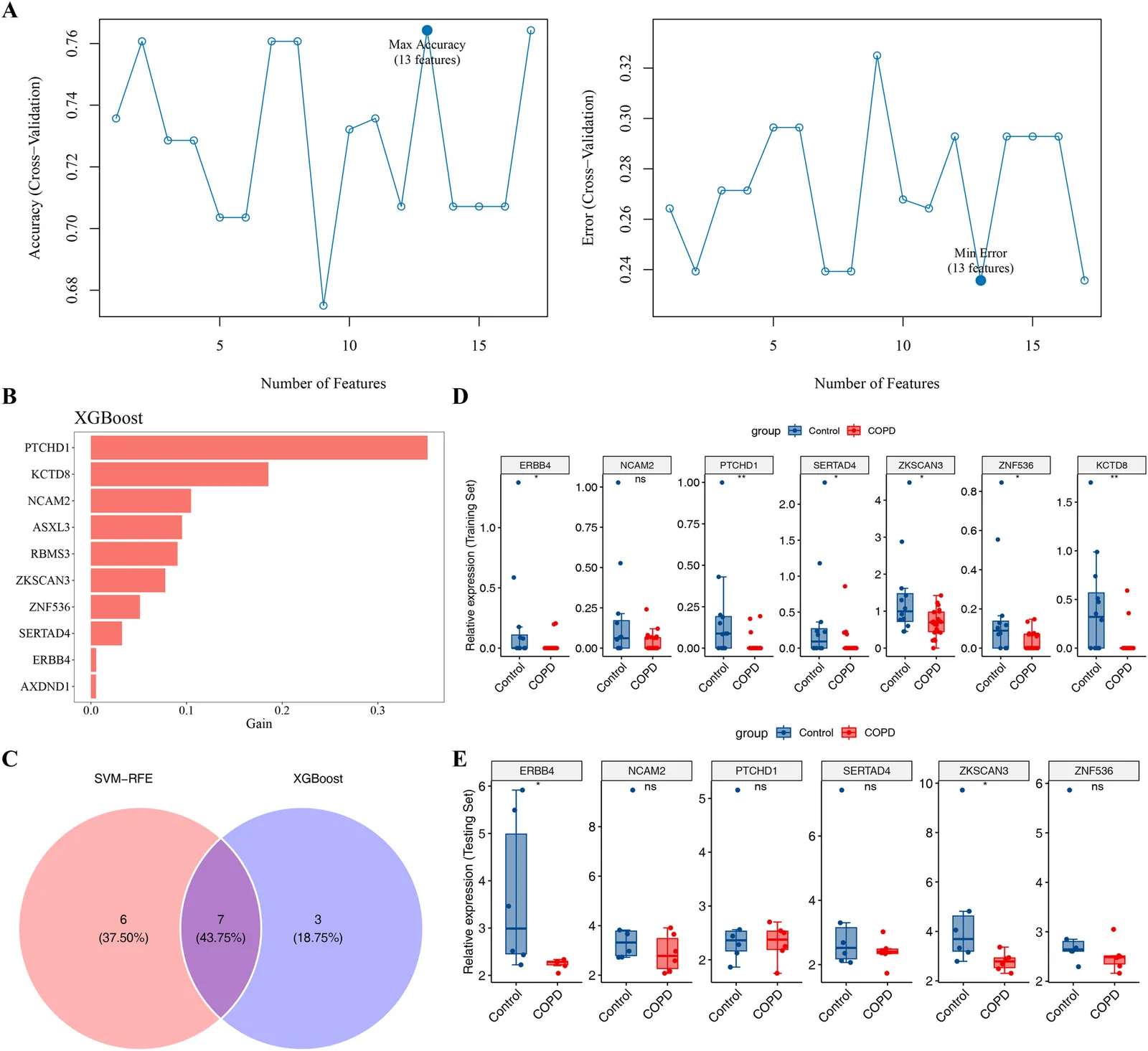
Machine learning-based screening and validation of diagnostic biomarkers. **(A)** Support vector machine-recursive feature elimination (SVM-RFE) identified 13 candidate genes with optimal classification performance. **(B)** XGBoost analysis showing the relative importance of candidate genes. **(C)** Venn diagram showing the intersection of genes selected by SVM-RFE and XGBoost, resulting in seven key genes. **(D)** Expression levels of the seven key genes in the training dataset. **(E)** Expression levels of the seven key genes in the validation dataset. ERBB4 and ZKSCAN3 were consistently and significantly downregulated in COPD samples and were defined as biomarkers. SVM-RFE, support vector machine-recursive feature elimination; XGBoost, extreme gradient boosting; COPD, chronic obstructive pulmonary disease.

### 3.4 Diagnostic performance of biomarkers

The nomogram showed that reduced ERBB4 and ZKSCAN3 expression was associated with an increased probability of COPD (Figure 4A). As an example, a total nomogram score of 177 points corresponded to a predicted COPD probability of 0.874. DCA indicated that the nomogram provided the greatest net benefit and favorable clinical utility relative to treat-all and treat-none strategies (Figure 4B). However, because of the limited sample size and absence of independent validation, the predictive performance should be interpreted cautiously. The calibration plot demonstrated good agreement, supported by a Hosmer-Lemeshow test with *P* > 0.05 (Figure 4C), suggesting reliable model prediction.

**Figure 4:**
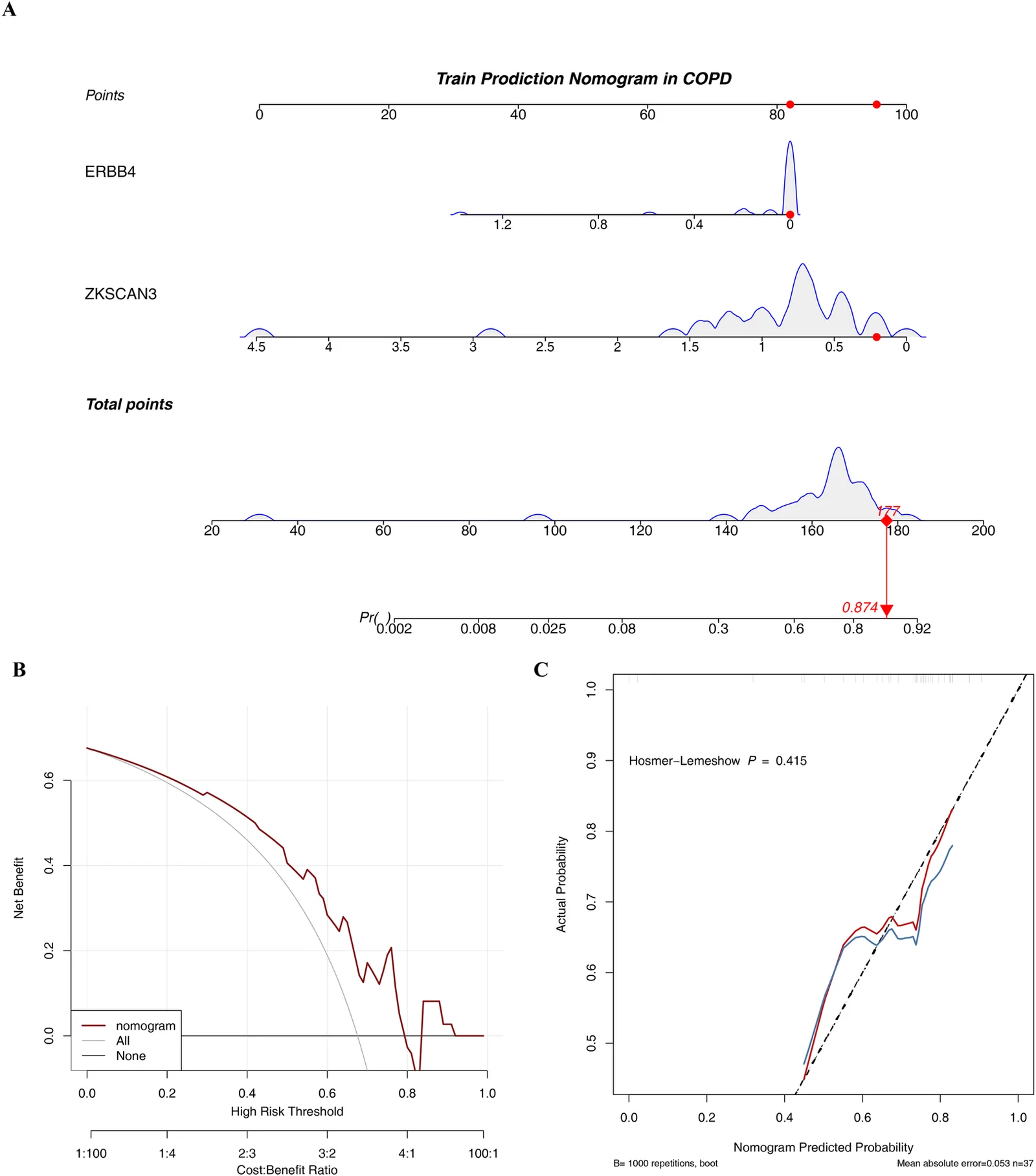
Construction and evaluation of the diagnostic nomogram based on ERBB4 and ZKSCAN3. **(A)** Nomogram for predicting the probability of COPD based on ERBB4 and ZKSCAN3 expression. A representative total score of 177 points corresponded to a predicted COPD probability of 0.874. **(B)** Decision curve analysis showing the clinical net benefit of the nomogram compared with treat-all and treat-none strategies. **(C)** Calibration curve evaluating the agreement between predicted and observed COPD probability. COPD, chronic obstructive pulmonary disease.

### 3.5 Functional analysis of biomarkers

Gene-gene interaction (GGI) network analysis placed ERBB4 and ZKSCAN3 at hub positions, with close connections to CFL1, GAPDH, HSP90AA1, and other genes (Figure 5). Functional enrichment of neighboring genes in this network suggested involvement in positive regulation of peptidyl-tyrosine phosphorylation, erythroblastosis oncogene B (ErbB) signaling, regulation of protein tyrosine kinase activity, and protein kinase B signaling. To investigate whether ERBB4 and ZKSCAN3 expression differences might reflect changes in immune cell composition, we performed ssGSEA cell-type deconvolution on GSE248493. COPD samples showed significantly higher Activated CD8T cell scores (*P* = 0.032) and lower Eosinophil (*P* = 0.025), Immature dendritic cell (*P* = 0.002), and Macrophage (*P* = 0.035) scores compared to controls. ZKSCAN3 was significantly positively correlated with Activated CD8T cells (*r* = 0.411, *P* = 0.012), whereas ERBB4 showed no significant correlation with any immune cell type. The observed gene expression differences may therefore partly reflect immune cell composition changes.

**Figure 5:**
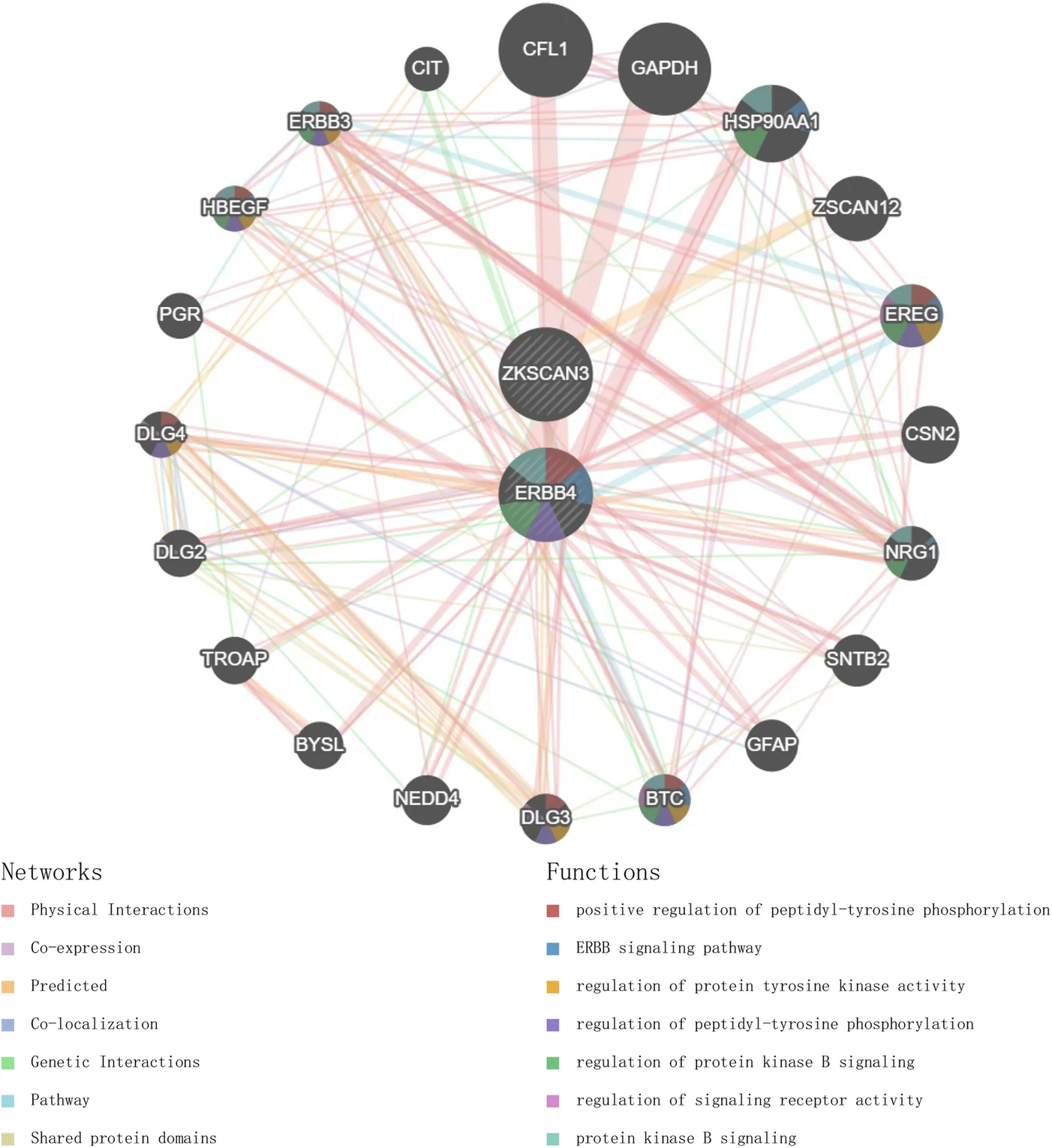
Gene-gene interaction network of ERBB4 and ZKSCAN3. GeneMANIA analysis was used to construct a gene-gene interaction network for the two biomarkers. ERBB4 and ZKSCAN3 occupied central positions in the network and were closely connected with functionally related genes, including CFL1, GAPDH, and HSP90AA1. Functional enrichment of neighboring genes suggested associations with positive regulation of peptidyl-tyrosine phosphorylation, ErbB signaling, regulation of protein tyrosine kinase activity, and protein kinase B signaling.

### 3.6 Pathways significantly enriched by biomarkers

For ERBB4, GSEA identified four significantly enriched pathways (|NES| >1, *P* < 0.05) (Figure 6A; Supplementary Table S7): ribosome, olfactory transduction, neuroactive ligand-receptor interaction, and retinol metabolism. For ZKSCAN3, four pathways also reached significance (|NES| >1, *P* < 0.05) (Figure 6B), including ribosome, spliceosome, extracellular matrix (ECM)-receptor interaction, and olfactory transduction. Ribosome and olfactory transduction were shared by both biomarkers.

**Figure 6:**
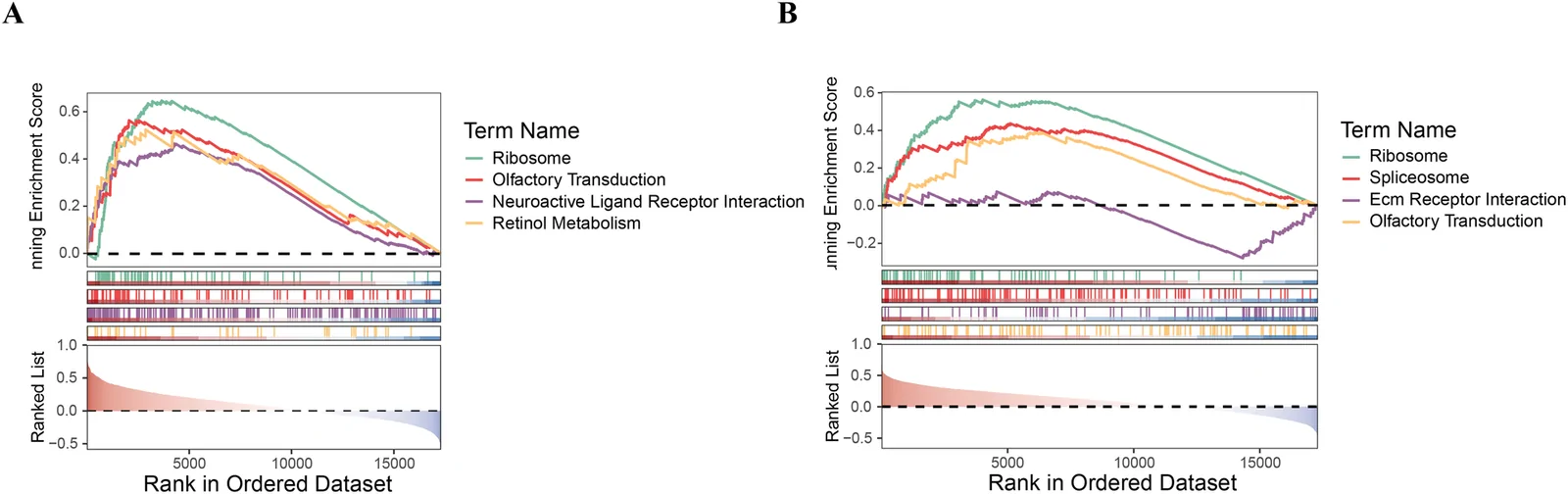
Gene set enrichment analysis of ERBB4 and ZKSCAN3. **(A)** Significantly enriched KEGG pathways associated with ERBB4 expression in GSE248493, including ribosome, olfactory transduction, neuroactive ligand-receptor interaction, and retinol metabolism. **(B)** Significantly enriched KEGG pathways associated with ZKSCAN3 expression, including ribosome, spliceosome, extracellular matrix-receptor interaction, and olfactory transduction. Pathways were considered significant at |normalized enrichment score| >1 and *P* < 0.05. KEGG, Kyoto Encyclopedia of Genes and Genomes; ECM, extracellular matrix.

### 3.7 Construction of mRNA-miRNA regulatory network

The mRNA-miRNA regulatory analysis predicted 75 miRNAs (Figure 7). ERBB4 was predicted to be targeted by 71 miRNAs, including hsa-miR-299-3p, hsa-miR-181d-5p, and hsa-miR-520g-3p. In contrast, ZKSCAN3 was predicted to interact with 4 miRNAs: hsa-miR-29a-3p, hsa-miR-29b-3p, hsa-miR-29c-3p, and hsa-miR-5094.

**Figure 7:**
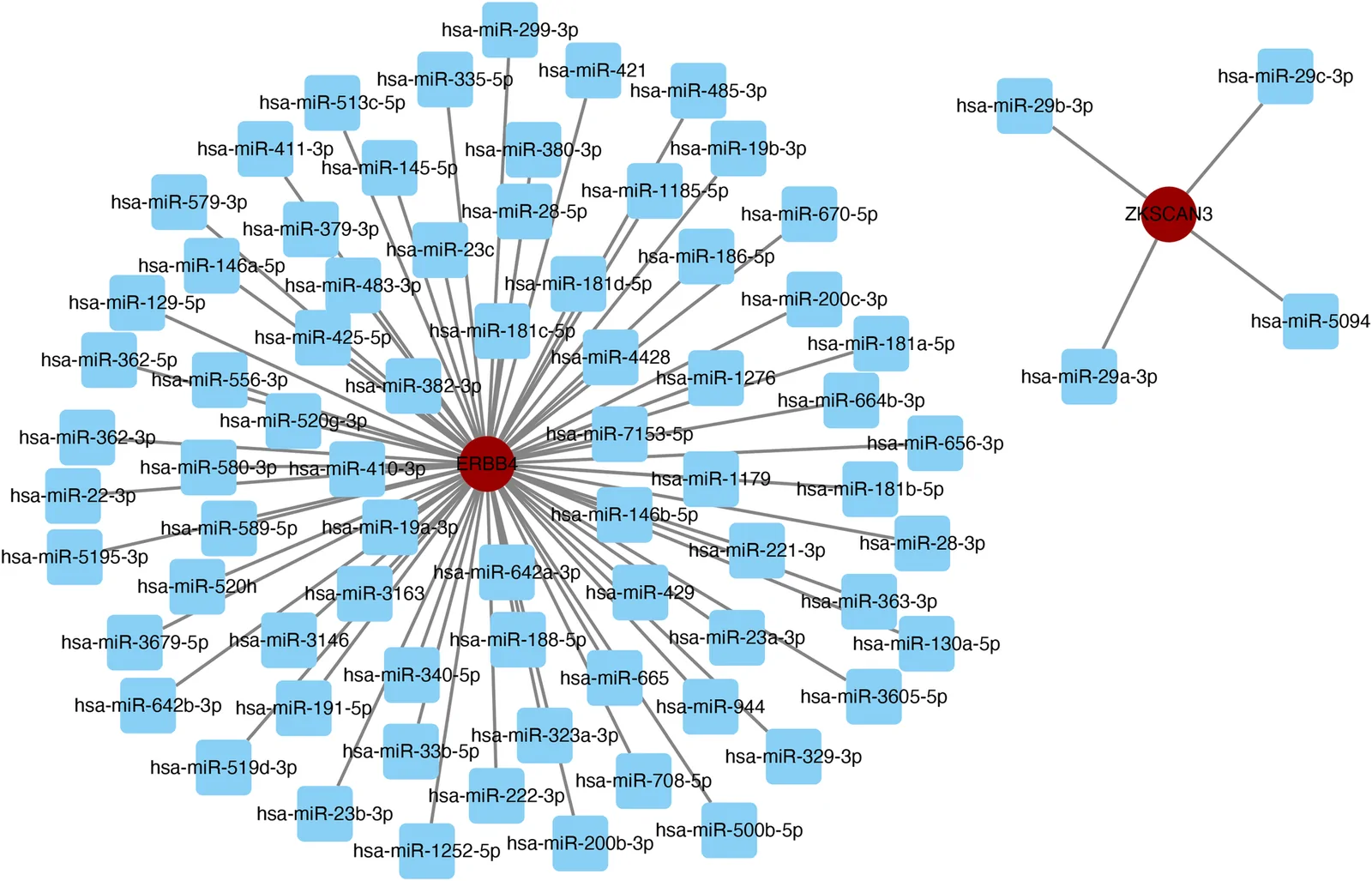
mRNA-miRNA regulatory network of ERBB4 and ZKSCAN3. The ENCORI database was used to predict miRNAs targeting the two biomarkers, and the regulatory network was visualized using Cytoscape. A total of 75 miRNAs were predicted, including 71 miRNAs targeting ERBB4 and four miRNAs targeting ZKSCAN3. Representative ERBB4-targeting miRNAs included hsa-miR-299-3p, hsa-miR-181d-5p, and hsa-miR-520g-3p, whereas ZKSCAN3 was predicted to interact with hsa-miR-29a-3p, hsa-miR-29b-3p, hsa-miR-29c-3p, and hsa-miR-5094. mRNA, messenger RNA; miRNA, microRNA.

### 3.8 Validation of biomarker expression by RT-qPCR

RT-qPCR in a small exploratory cohort (*n* = 5 per group) showed directionally consistent downregulation of ERBB4 and ZKSCAN3 in COPD samples (Figures 8A,B), supporting the bioinformatics findings.

**Figure 8:**
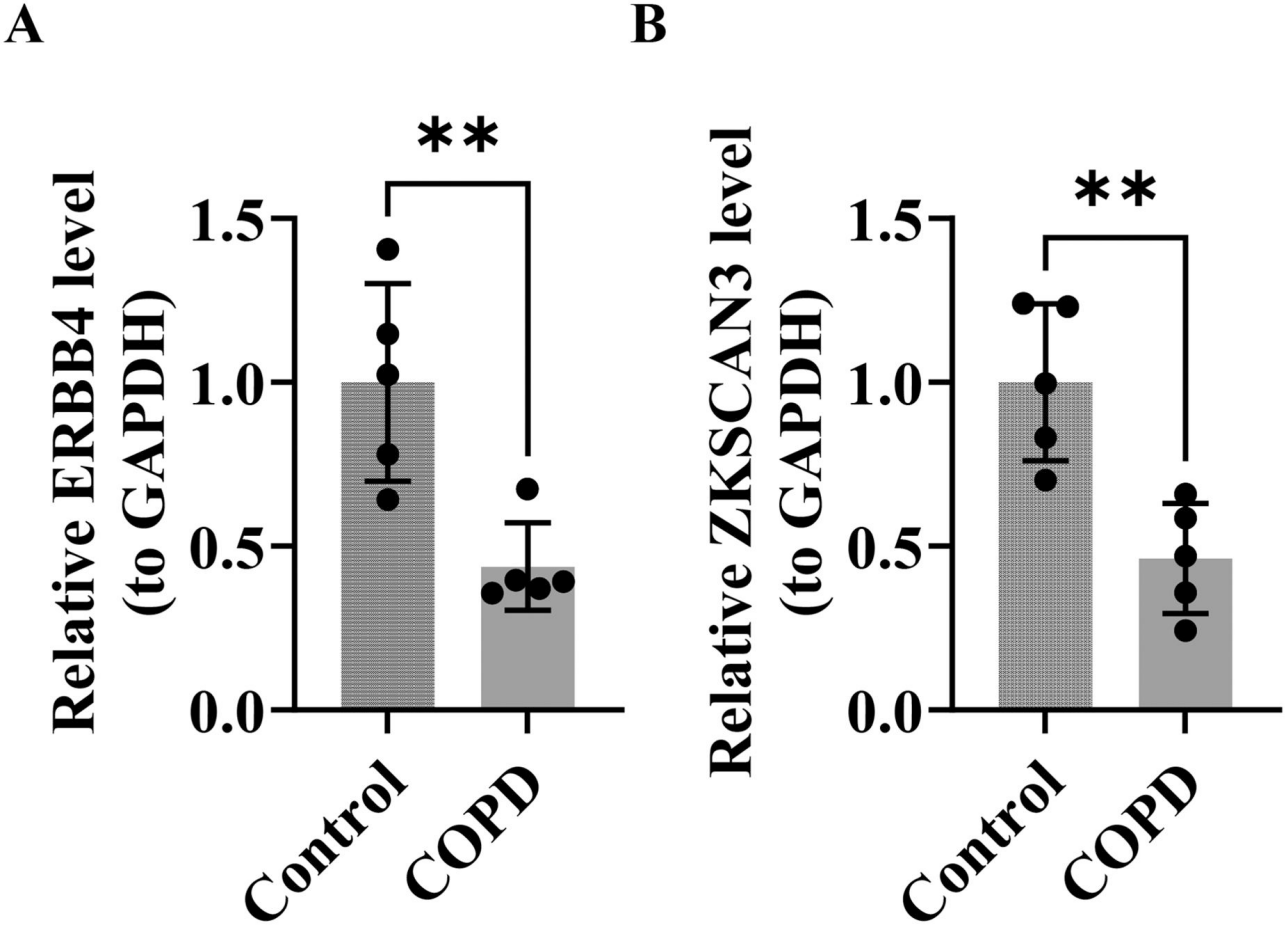
RT-qPCR validation of ERBB4 and ZKSCAN3 expression. **(A,B)** RT-qPCR analysis confirmed that, compared with control samples (*n* = 5), the expression levels of ERBB4 and ZKSCAN3 were significantly reduced in COPD samples (*n* = 5), consistent with the results of the bioinformatics analysis. Data points represent individual biological replicates. Bar graphs show mean ± SD. *P* < 0.05; *P* < 0.01 (Wilcoxon rank-sum test). RT-qPCR, reverse transcription quantitative polymerase chain reaction; COPD, chronic obstructive pulmonary disease.

## 4 Discussion

COPD is a highly heterogeneous disorder shaped by multiple pathogenic drivers, with persistent airflow limitation, chronic airway inflammation, and oxidative stress imbalance as its central features (27, 28). Se performs most of its biological functions through selenium metabolism and selenoprotein systems (9, 29). These proteins participate in ROS elimination, lipid peroxide reduction, inflammatory signaling, and immune regulation (11, 30), all of which are directly related to COPD pathology. Nevertheless, how selenoprotein networks are altered during COPD remains incompletely understood. Integrating selenium metabolism-related signatures with COPD transcriptomic profiles may therefore help identify redox-related molecular patterns and diagnostically useful targets. In this study, COPD datasets from GEO were analyzed together with the SMASP gene set. Differential expression analysis was combined with SVM-RFE and XGBoost to screen selenium-associated COPD biomarkers. We further applied a nomogram, GeneMANIA network analysis, GSEA, and regulatory network construction, followed by RT-qPCR validation, to investigate the potential mechanisms involved in selenoprotein-associated COPD progression. These findings provide a theoretical basis for early biomarker discovery and targeted therapeutic development in COPD.

The analysis first identified 235 DEGs in GEO COPD datasets. WGCNA then detected a key module related to selenium metabolism that contained 4,288 genes. Intersecting the module genes with DEGs generated 17 candidates, and seven core genes were subsequently selected by SVM-RFE and XGBoost. ERBB4 and ZKSCAN3 were finally confirmed as significantly downregulated biomarkers in COPD. A nomogram constructed from these two genes showed good calibration and favorable clinical net benefit. Several recent studies have integrated transcriptomic signatures with machine learning to construct gene signature-based nomograms for individualized prognosis and clinical decision-making in respiratory and other diseases (31, 32). These studies illustrate the growing utility of transcriptome-derived nomograms and highlight the importance of external validation, calibration, discrimination, and clinical utility assessment. In contrast, the nomogram developed in the present study was based on only two candidate genes in a small development cohort (*n* = 37) and was not externally validated. It should therefore be regarded as an exploratory visualization of the fitted logistic model rather than a clinically deployable prediction tool. Future studies should compare its performance with established COPD biomarkers and published transcriptomic models in larger independent cohorts.

GSEA showed that ERBB4 and ZKSCAN3 were both associated with ribosome and olfactory transduction pathways. The recurrent enrichment of olfactory transduction pathways warrants additional comment. Although olfactory receptors are classically associated with sensory function in the nasal epithelium, they are expressed in a variety of non-olfactory tissues including the lung, where they have been implicated in chemotaxis, smooth muscle regulation, and inflammatory responses. Their enrichment in PBMC transcriptomic datasets from COPD patients may reflect systemic transcriptional alterations rather than sensory function *per se*. This pathway's repeated enrichment across both biomarkers may indicate a broader, systemically detectable transcriptional signature of COPD rather than a lung-specific pathological mechanism. The mRNA-miRNA network predicted broader miRNA regulation of ERBB4, with 71 targeting miRNAs, while ZKSCAN3 was predicted to be controlled by four miRNAs, mainly members of the miR-29 family. Among the predicted miRNAs, the miR-29 family (miR-29a/b/c-3p) targeting ZKSCAN3 is of particular interest. miR-29 is a well-characterized regulator of pulmonary fibrosis, modulating the expression of multiple ECM-related genes. The predicted interaction suggests a potential ZKSCAN3-miR-29-ECM regulator*y* axis that may contribute to airway remodeling in COPD. For ERBB4, miR-193a-3p, identified in our network, has been experimentally validated as a direct ERBB4-targeting miRNA in lung cancer, consistent with a post-transcriptional regulatory mechanism for ERBB4 downregulation in COPD. These computational predictions, while informative, require experimental confirmation. RT-qPCR results were consistent with the computational analyses and verified the decreased expression of ERBB4 and ZKSCAN3 in COPD samples. However, The RT-qPCR validation was performed using five COPD and five control samples. Although the results were directionally consistent with the computational analyses, this sample size is insufficient to establish the robustness, reproducibility, or clinical utility of the biomarker signature. Larger independent cohorts are required for definitive validation.

The selenium-related module provides a framework for interpreting redox-associated mechanisms in COPD. Selenium deficiency is frequently observed in COPD patients and is closely associated with disease progression (9, 11). Glutathione peroxidase 4 (GPX4), an essential selenoprotein, removes peroxidized phospholipids and prevents lipid peroxide accumulation; loss of this activity can drive excessive lipid peroxidation and emphysema development (31). In the present analysis, COPD samples exhibited markedly lower SMASP scores, suggesting an overall weakening of selenoprotein-mediated antioxidant defense. This finding is compatible with the established redox imbalance in COPD and may reflect reduced selenium-dependent antioxidant capacity, which could favor lipid peroxidation and ferroptosis-related epithelial injury (5, 9, 33). Beyond selenium-dependent antioxidant enzymes, recent studies have highlighted the importance of broader redox-regulatory networks in pulmonary diseases. A recent study reported that glutathione reductase (GSR) deficiency impaired intracellular glutathione recycling, resulting in increased ROS accumulation and activation of the TGF-*β*/Smad2 pathway, thereby promoting epithelial-mesenchymal transition and fibroblast activation during pulmonary fibrosis (34). These findings further support the concept that disruption of antioxidant homeostasis represents a common mechanism underlying chronic lung injury. Therefore, the reduced SMASP score observed in our study may reflect a systemic impairment of antioxidant defense networks rather than isolated alterations in selenium-related genes.

GO enrichment analysis suggests that these candidate genes may be involved in biological processes such as neural crest cell migration, mesenchymal cell migration and stem cell development; these processes are potentially linked to the epithelial-mesenchymal transition (EMT) that occurs during airway remodelling. These findings suggest that selenium-related transcriptional changes may be involved in epithelial plasticity and extracellular matrix remodelling—both of which are key factors leading to irreversible airflow limitation (35). It has been reported in the literature that, in smoking-related COPD, EMT can induce airway epithelial cells to adopt a mesenchymal phenotype, enhancing their migratory and invasive capabilities, and promoting airway fibrosis and fixed airflow obstruction (35). The enrichment of peroxisome proliferator-activated receptors (PPARs) within the MF category may also be biologically significant. Previous studies have shown that PPAR*γ* expression levels are reduced in COPD airway epithelial cells, whilst PPAR*γ* agonists can attenuate the inflammatory phenotype induced by cigarette smoke and enhance the therapeutic response to corticosteroids (36). PPAR*α* may also alleviate COPD-associated skeletal muscle dysfunction by regulating fatty acid metabolism, reducing ROS production and inhibiting NF-*κ*B-driven inflammatory transcription (37).

KEGG analysis suggests that the PI3K-Akt signalling pathway—a signalling axis known to be involved in COPD inflammation and oxidative stress—may be associated with the candidate genes identified in this study. The literature indicates that cigarette smoke can activate the PI3K-Akt signalling pathway in alveolar macrophages and airway epithelial cells, promoting the release of pro-inflammatory cytokines (including TNF-α and IL-1β) and affecting the M1/M2 macrophage polarisation balance (38). The enrichment of nitrogen metabolism and alanine-aspartate-glutamate metabolism further suggests that amino acid metabolic reprogramming may be associated with redox imbalance in COPD. Taken together, these candidate genes are associated with the antioxidant function of selenoproteins, airway remodelling, inflammation and metabolic disorders via the EMT, PPAR and PI3K-Akt signalling pathways, suggesting that they may be of value for exploration in downstream biomarker screening. The only pathway that remained significant after multiple-testing correction was “Proteoglycans in cancer.” This pathway is biologically relevant to COPD, as proteoglycans are structural components of the extracellular matrix and their dysregulation contributes to airway remodeling and fibrosis. The remaining enriched pathways—including ribosome, ECM-receptor interaction, and neuroactive ligand-receptor interaction—are discussed as exploratory observations that merit further investigation. The mechanistic links between ERBB4/ZKSCAN3 and selenium biology discussed here are derived from computational associations and published literature, not from functional experiments performed in this study. We therefore interpret these connections as biologically plausible hypotheses rather than established mechanisms. Future studies with selenium supplementation, gene-specific manipulation, and protein-level measurements in COPD-relevant models are required to determine whether ERBB4 or ZKSCAN3 is directly regulated by selenium status or participates in selenium-dependent pathways.

Comprehensive bioinformatics screening ultimately highlighted ERBB4 and ZKSCAN3 as diagnostic biomarkers for COPD. ERBB4 belongs to the ErbB receptor tyrosine kinase family and has important roles in lung development. The neuregulin-ErbB4 signaling axis regulates alveolar type II (ATII) epithelial cell maturation and pulmonary surfactant synthesis (39). During late gestation, the ErbB4-Jma isoform is strongly upregulated in fetal rat ATII cells and promotes ATII differentiation as well as Sftpb expression (40). In adult rat lung epithelium, ERBB4 contributes to basal surfactant phospholipid synthesis, and its downregulation markedly impairs endogenous surfactant production (41). These developmental and epithelial functions help explain the significant decrease in ERBB4 observed in COPD. Because COPD involves ATII cell dysfunction and altered surfactant metabolism, reduced ERBB4 may indicate weakened alveolar epithelial repair capacity. Such downregulation may be closely related to persistent epithelial injury, disrupted repair homeostasis, suppressed ATII maturation, and impaired surfactant-related recovery during COPD progression (40–42).

ERBB4 is subject to isoform-specific regulation through alternative splicing. The Jma isoform contains a TACE cleavage site that permits proteolytic release of the intracellular 4ICD domain, which can act as a nuclear transcriptional co-regulator. In contrast, the Jmb isoform lacks this site and does not undergo the same post-translational processing (43). This isoform heterogeneity may explain the context-dependent and disease stage-specific functions of ERBB4 in COPD. GSEA linked ERBB4 to ribosome and neuroactive ligand-receptor interaction pathways. Ribosome enrichment is consistent with the substantial protein synthesis demand of ATII cells during surfactant protein production (39). Disturbed ribosomal activity may lead to impaired protein synthesis and endoplasmic reticulum stress, which is known to promote airway epithelial apoptosis in COPD (44). In addition, ERBB4 was predicted to be regulated by 71 miRNAs. Previous lung cancer evidence showed that miR-193a-3p directly targets the ERBB4 3'-UTR and suppresses its translation (45). Thus, ERBB4 reduction in COPD may reflect remodeling of post-transcriptional regulatory networks rather than transcriptional repression alone.

ZKSCAN3 (zinc finger with KRAB and SCAN domains 3) is known as a transcriptional repressor of autophagy and lysosomal biogenesis. It suppresses more than 60 autophagy-related genes, including Map1lc3b, Ulk1, and Wipi2, and counteracts TFEB-mediated transcription (46). In pancreatic cancer cells, ZKSCAN3 deletion markedly increases LC3-II, a classical autophagy marker, and enhances proliferation, migration, and invasion, whereas ZKSCAN3 overexpression reverses these phenotypes (47). However, in several solid tumors, high ZKSCAN3 expression promotes tumor progression by increasing CCND1, EGFR, and VEGF, indicating that its biological effects are strongly tissue- and context-dependent (48).

In this study, ZKSCAN3 expression was significantly reduced in COPD samples. Its enrichment in ECM-receptor interaction, spliceosome, and ribosome pathways suggests that ZKSCAN3 may have multiple pathological implications in COPD. Reduced ZKSCAN3 may, on one side, permit excessive autophagy and thereby contribute to alveolar epithelial senescence and apoptosis. On the other side, loss of ZKSCAN3-mediated repression may facilitate ECM remodeling programs and promote progressive airway fibrosis. The predicted regulation of ZKSCAN3 by the miR-29 family (miR-29a/b/c-3p) is particularly noteworthy because this miRNA family is a recognized regulator of pulmonary fibrosis (49). These results suggest that a ZKSCAN3-miR-29-ECM axis may participate in COPD-related airway remodeling. Given the established involvement of miR-29 in fibrotic remodeling, reduced ZKSCAN3 may interact with miR-29-driven ECM programs to influence airway structural changes (47–49). Spliceosome pathway enrichment is also consistent with the transcriptional regulatory nature of ZKSCAN3. As a KRAB domain-containing zinc finger protein, ZKSCAN3 can recruit the KAP1/SETDB1 co-repressor complex to target promoters, inducing heterochromatin formation and broad epigenetic remodeling (50).

Clinical studies have explored selenium supplementation in COPD, and its potential benefits may depend partly on increased expression or activity of selenoproteins such as GPXs and thioredoxin reductases, which strengthen antioxidant defense (9, 11). In the key functional module identified here, genes showed strong positive correlations with selenoprotein expression (|r| > 0.3, *P* < 0.001). This observation suggests that ERBB4 and ZKSCAN3 may represent important nodes within the selenoprotein-related regulatory network, however, the present associations do not support selenium supplementation or ERBB4/ZKSCAN3-directed treatment. Any therapeutic relevance must be tested in controlled biochemical, cellular, animal, and clinical studies. Exogenous selenium may indirectly influence these molecules by reshaping selenoprotein profiles. This possibility is in line with NHANES findings that dietary selenium intake is inversely associated with COPD risk (OR = 0.80) (9) and with reports that intravenous selenium can reduce mechanical ventilation duration in severe COPD (11). The reduced SMASP scores in COPD samples further imply exhaustion of selenoprotein-dependent antioxidant defenses during disease progression. Alternatively, direct modulation of ERBB4 and ZKSCAN3 could be explored therapeutically. ERBB4 agonists such as Neuregulin-1β can promote ATII cell proliferation and surfactant synthesis in lung injury models (40, 42), potentially improving defective alveolar epithelial repair in COPD. Activation of ZKSCAN3 may lessen epithelial apoptosis by restraining excessive autophagy and may also attenuate airway fibrosis through the ZKSCAN3-miR-29-ECM regulator*y* axis (49). However, the isoform-specific biology of ERBB4, in which Jma produces the nuclear 4ICD cofactor whereas Jmb does not (43), and the context-dependent dual functions of ZKSCAN3 (47, 48) indicate that targeted interventions will require careful specificity and disease-context optimization. Based on the associations identified in this study, we propose that selenium supplementation, ERBB4 modulation, and ZKSCAN3 modulation merit investigation in future mechanistic studies. However, these remain speculative hypotheses requiring direct experimental confirmation in COPD-relevant models.

By integrating differential expression profiling, WGCNA, and machine learning, this study identified and validated ERBB4 and ZKSCAN3 as potential diagnostic biomarkers for COPD. Both genes were significantly downregulated in COPD specimens. The nomogram based on these two genes showed moderate discrimination in the development cohort but was not externally validated. It should be regarded as an exploratory research tool rather than a clinically deployable model, and its incremental diagnostic value over existing COPD biomarkers remains to be determined. Although transcriptomic approaches provide valuable insights into COPD-associated molecular alterations, mRNA signatures may not always fully represent downstream protein-level changes. Recent serum proteomic studies have identified circulating protein biomarkers associated with pulmonary fibrosis (51), demonstrating the potential value of proteome-based strategies for disease classification and prediction. Integrating selenium-related transcriptomic signatures with proteomic profiles and clinical variables may therefore provide a more comprehensive framework for future COPD biomarker development and improve the translational applicability of predictive models.

Several limitations should be considered. First, although RT-qPCR confirmed differential expression of the two candidate biomarkers, the clinical sample size was small and multi-center external validation was not performed. Although we attempted to validate the biomarkers in an independent GEO dataset (GSE42057), neither ERBB4 nor ZKSCAN3 was available in that dataset's expression matrix, precluding this analysis. The diagnostic performance of ERBB4 and ZKSCAN3 should therefore be compared with previously published COPD transcriptomic signatures in future studies. The present data are insufficient to establish superiority over existing biomarkers. Second, the small sample size represents an important limitation for machine learning-based biomarker discovery. Although SVM-RFE and XGBoost were used for exploratory feature prioritization, the limited number of samples may increase the risk of overfitting and reduce feature-selection stability. Repeated nested resampling attempts showed insufficient stability, and therefore the identified biomarkers require validation in larger independent cohorts before clinical application. Third, clinical covariates such as age, sex, smoking status and intensity, GOLD stage, lung function (FEV1, FVC, FEV1/FVC), disease stability, medication use, comorbidities, and infection status were not available in the public GEO metadata. We were therefore unable to adjust for these potential confounders. Although cell-type deconvolution suggested that immune composition differences contribute to the observed expression changes, definitive adjustment for leukocyte composition was not feasible. These limitations should be considered in interpreting the results. Fouth, the selenium specificity of ERBB4 and ZKSCAN3 has not been established in this study. We did not measure serum or tissue selenium concentrations, selenoprotein levels, GPX activity, or response to selenium manipulation. Therefore, these genes should be considered as candidates associated with selenium-related transcriptional networks in COPD, rather than validated selenium-metabolism genes. Future studies incorporating selenium supplementation, gene-specific manipulation, and protein-level measurements are required to confirm selenium specificity. Fifth, the upstream and downstream molecular networks through which ERBB4 and ZKSCAN3 influence COPD pathogenesis remain to be systematically defined. Sixth, the biological functions of these genes and the efficacy of targeted intervention have not yet been verified in cell or animal models. Seventh, PBMCs were used for public transcriptomic analysis, the PBMC expression findings cannot be directly extrapolated to lung epithelial cells, including alveolar type II cells. Confirmatory analyses using lung tissue specimens are required. Eighth, because the study compared established COPD cases with controls, we cannot claim early diagnostic utility. Prospective studies in at-risk populations are needed to evaluate the predictive potential of these biomarkers. Ninth, the present work was restricted to transcriptional bioinformatics analysis, without protein-level verification or functional rescue experiments; therefore, the molecular mechanisms and biological roles of the identified targets require further confirmation.

Future work should address these limitations. Larger clinical cohorts covering different COPD subtypes, such as emphysema-predominant and chronic bronchitis-predominant phenotypes, are needed to further evaluate biomarker sensitivity and specificity. In addition, *in vitro* experiments using pulmonary epithelial cells and *in vivo* studies based on cigarette smoke-induced COPD mouse models should be performed to clarify how ERBB4 and ZKSCAN3 regulate pulmonary inflammation, oxidative stress homeostasis, and epithelial repair during COPD progression.

## Data Availability

The datasets presented in this study can be found in online repositories. The names of the repository/repositories and accession number(s) can be found in the article/Supplementary Material. Fixed random seeds were used throughout: set.seed(13) for SVM-RFE; set.seed(1985) and set.seed(411) for XGBoost; set.seed(123) for nomogram DCA and WGCNA. Package versions are provided in the methods and analysis scripts.
