# Supplementary material for: Identification of candidate selenium-associated transcriptomic biomarkers in chronic obstructive pulmonary disease: an integrated bioinformatics and expression validation study
Source: Front Med (Lausanne). 2026 Sep 30;13:1909444. doi: 10.3389/fmed.2026.1909444 (PMC13662921; doi:10.3389/fmed.2026.1909444)
Supplement: Supplementary file 1 [file Datasheet1.zip › Supplementary Materials/02_WGCNA/02.clustering.pdf]

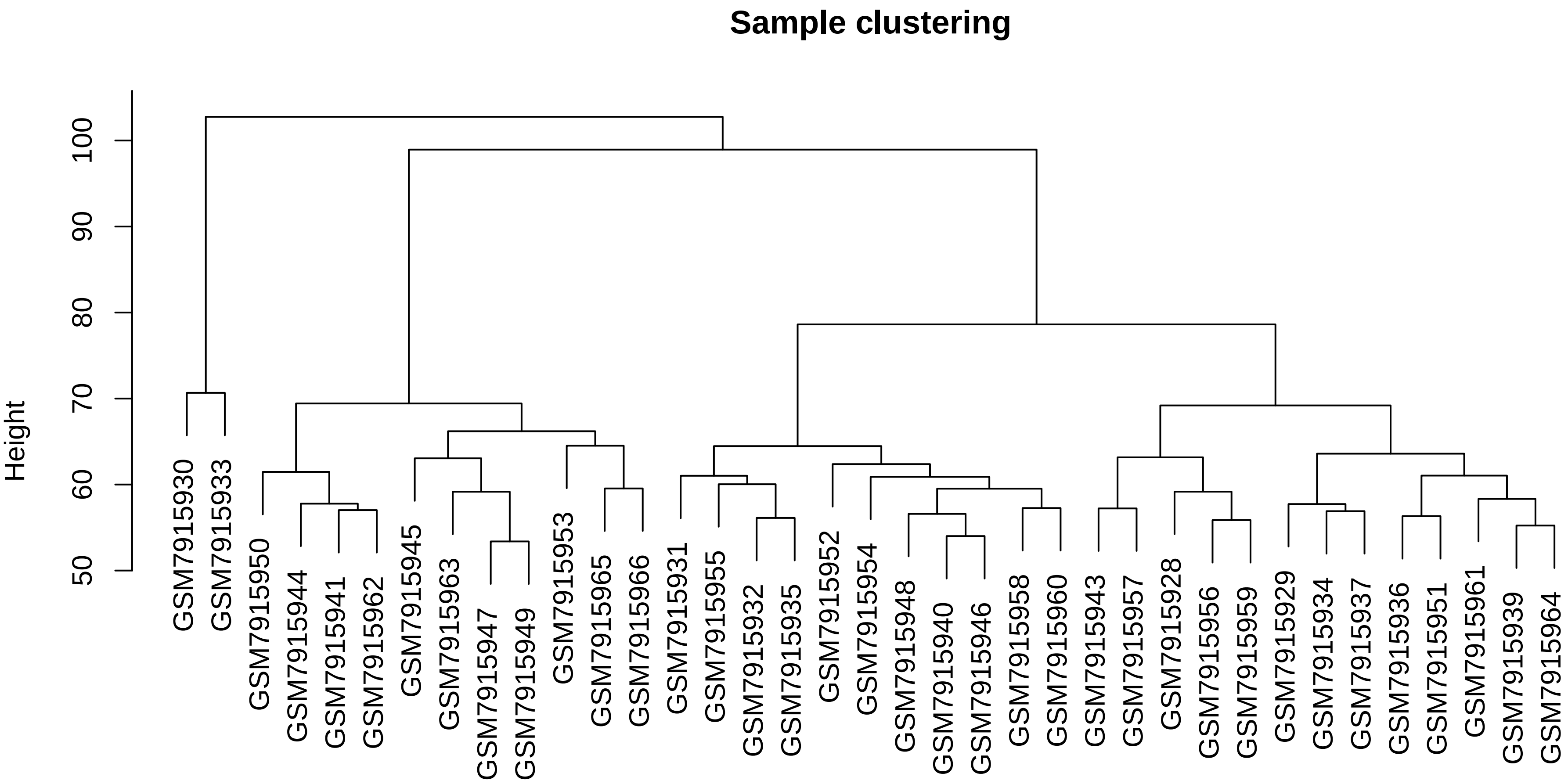

Sample clustering
Height
100
90
80
70
60
50
GSM7915930
GSM7915933
GSM7915950
GSM7915944
GSM7915941
GSM7915962
GSM7915945
GSM7915963
GSM7915947
GSM7915949
GSM7915953
GSM7915965
GSM7915966
GSM7915931
GSM7915955
GSM7915932
GSM7915935
GSM7915952
GSM7915954
GSM7915948
GSM7915940
GSM7915946
GSM7915958
GSM7915960
GSM7915943
GSM7915957
GSM7915928
GSM7915956
GSM7915959
GSM7915929
GSM7915934
GSM7915937
GSM7915936
GSM7915951
GSM7915961
GSM7915939
GSM7915964
