# Supplementary material for: Identification of candidate selenium-associated transcriptomic biomarkers in chronic obstructive pulmonary disease: an integrated bioinformatics and expression validation study
Source: Front Med (Lausanne). 2026 Sep 30;13:1909444. doi: 10.3389/fmed.2026.1909444 (PMC13662921; doi:10.3389/fmed.2026.1909444)
Supplement: Supplementary file 1 [file Datasheet1.zip › Supplementary Materials/02_WGCNA/03.software.pdf]

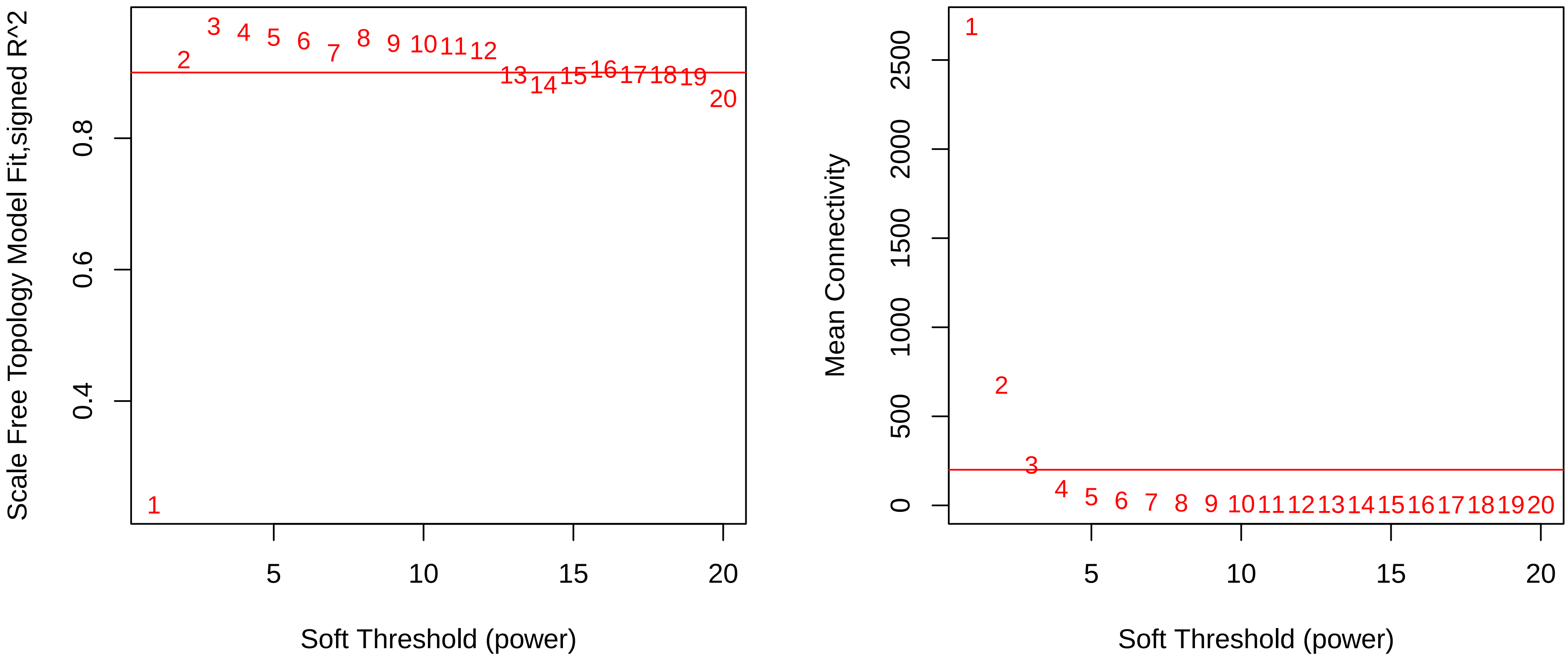
Scale Free Topology Model Fit,signed R^2
0.4
0.6
0.8
1
2
3
4
5
6
7
8
9
10
11
12
13
14
15
16
17
18
19
20
5
10
15
20
Soft Threshold (power)
Mean Connectivity
0
500
1000
1500
2000
2500
1
2
3
4
5
6
7
8
9
10
11
12
13
14
15
16
17
18
19
20
5
10
15
20
Soft Threshold (power)
