# Supplementary material for: Identification of candidate selenium-associated transcriptomic biomarkers in chronic obstructive pulmonary disease: an integrated bioinformatics and expression validation study
Source: Front Med (Lausanne). 2026 Sep 30;13:1909444. doi: 10.3389/fmed.2026.1909444 (PMC13662921; doi:10.3389/fmed.2026.1909444)
Supplement: Supplementary file 1 [file Datasheet1.zip › Supplementary Materials/02_WGCNA/06.labeledHeatmap.pdf]

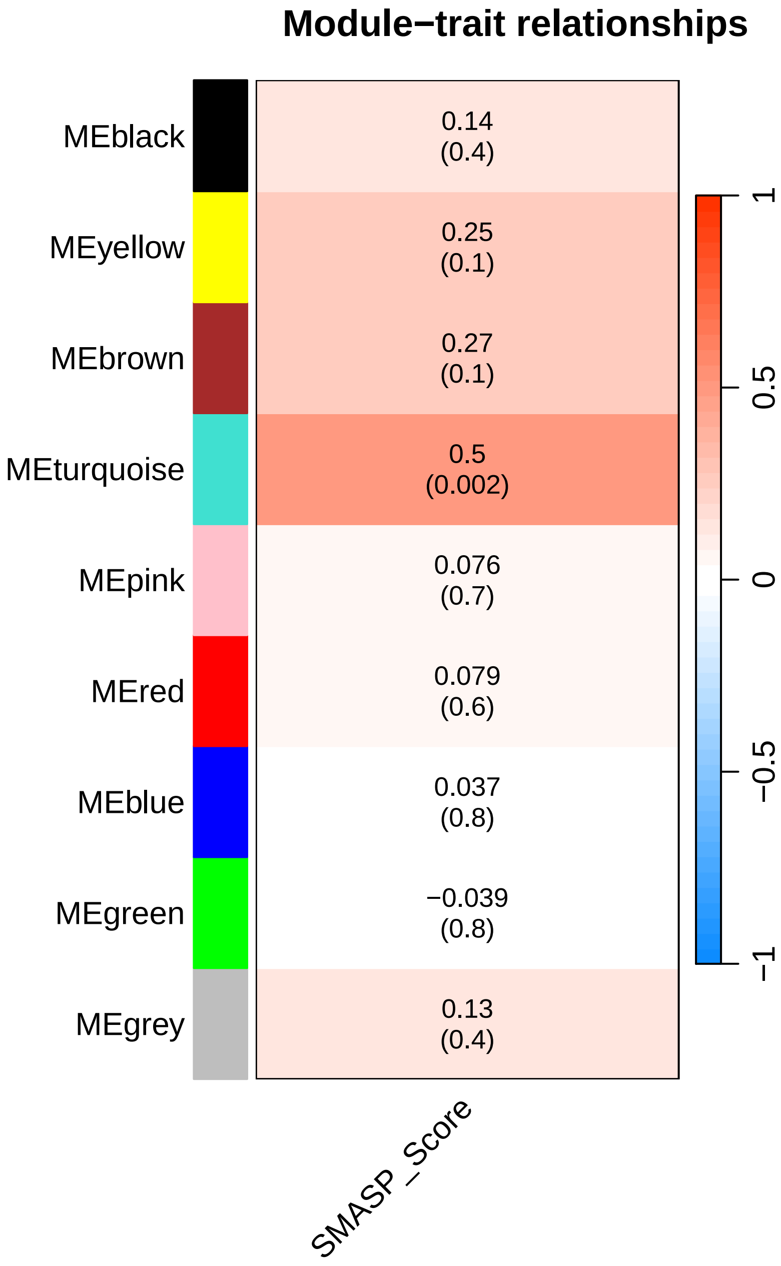
Module−trait relationships
MEblack
MEyellow
MEbrown
MEturquoise
MEpink
MEred
MEblue
MEgreen
MEgrey
0.14
(0.4)
0.25
(0.1)
0.27
(0.1)
0.5
(0.002)
0.076
(0.7)
0.079
(0.6)
0.037
(0.8)
−0.039
(0.8)
0.13
(0.4)
1
0.5
0
−0.5
−1
SMASP_Score
