# Supplementary material for: Identification of candidate selenium-associated transcriptomic biomarkers in chronic obstructive pulmonary disease: an integrated bioinformatics and expression validation study
Source: Front Med (Lausanne). 2026 Sep 30;13:1909444. doi: 10.3389/fmed.2026.1909444 (PMC13662921; doi:10.3389/fmed.2026.1909444)
Supplement: Supplementary file 1 [file Datasheet1.zip › Supplementary Materials/02_WGCNA/08.MM_GS_turquoise.pdf]

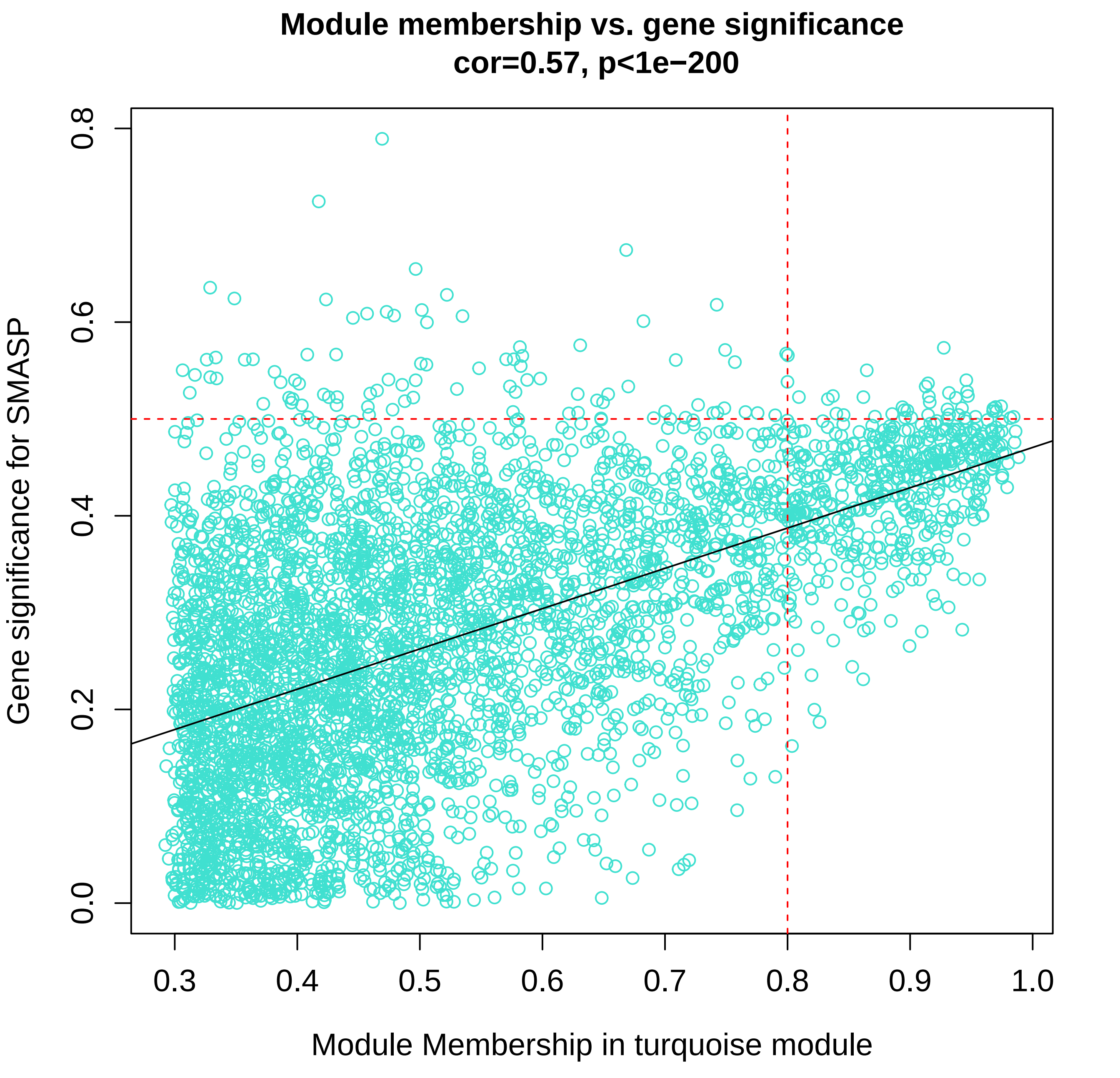
Module membership vs. gene significance
cor=0.57, p<1e−200
Gene significance for SMASP
Module Membership in turquoise module
0.0
0.2
0.4
0.6
0.8
0.3
0.4
0.5
0.6
0.7
0.8
0.9
1.0
