# Supplementary material for: Identification of candidate selenium-associated transcriptomic biomarkers in chronic obstructive pulmonary disease: an integrated bioinformatics and expression validation study
Source: Front Med (Lausanne). 2026 Sep 30;13:1909444. doi: 10.3389/fmed.2026.1909444 (PMC13662921; doi:10.3389/fmed.2026.1909444)
Supplement: Supplementary file 1 [file Datasheet1.zip › Supplementary Materials/07_Nomogram/01.Nomogram.pdf]

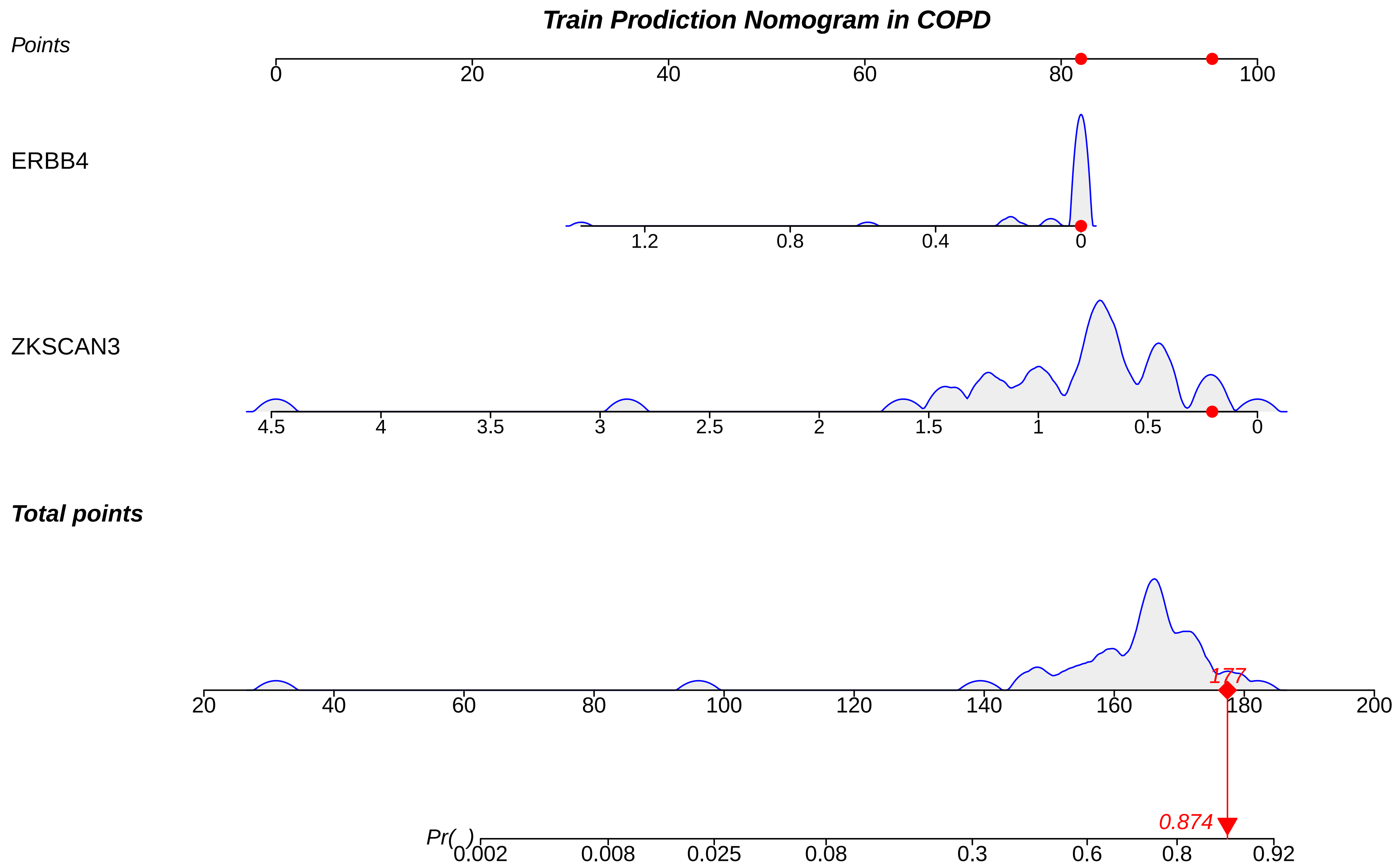
Train Prodiction Nomogram in COPD
Points
0
20
40
60
80
100
ERBB4
1.2
0.8
0.4
0
ZKSCAN3
4.5
4
3.5
3
2.5
2
1.5
1
0.5
0
Total points
177
20
40
60
80
100
120
140
160
180
200
0.874
Pr( )
0.002
0.008
0.025
0.08
0.3
0.6
0.8
0.92
