# Supplementary material for: Identification of candidate selenium-associated transcriptomic biomarkers in chronic obstructive pulmonary disease: an integrated bioinformatics and expression validation study
Source: Front Med (Lausanne). 2026 Sep 30;13:1909444. doi: 10.3389/fmed.2026.1909444 (PMC13662921; doi:10.3389/fmed.2026.1909444)
Supplement: Supplementary file 1 [file Datasheet1.zip › Supplementary Materials/07_Nomogram/03.DCA.pdf]

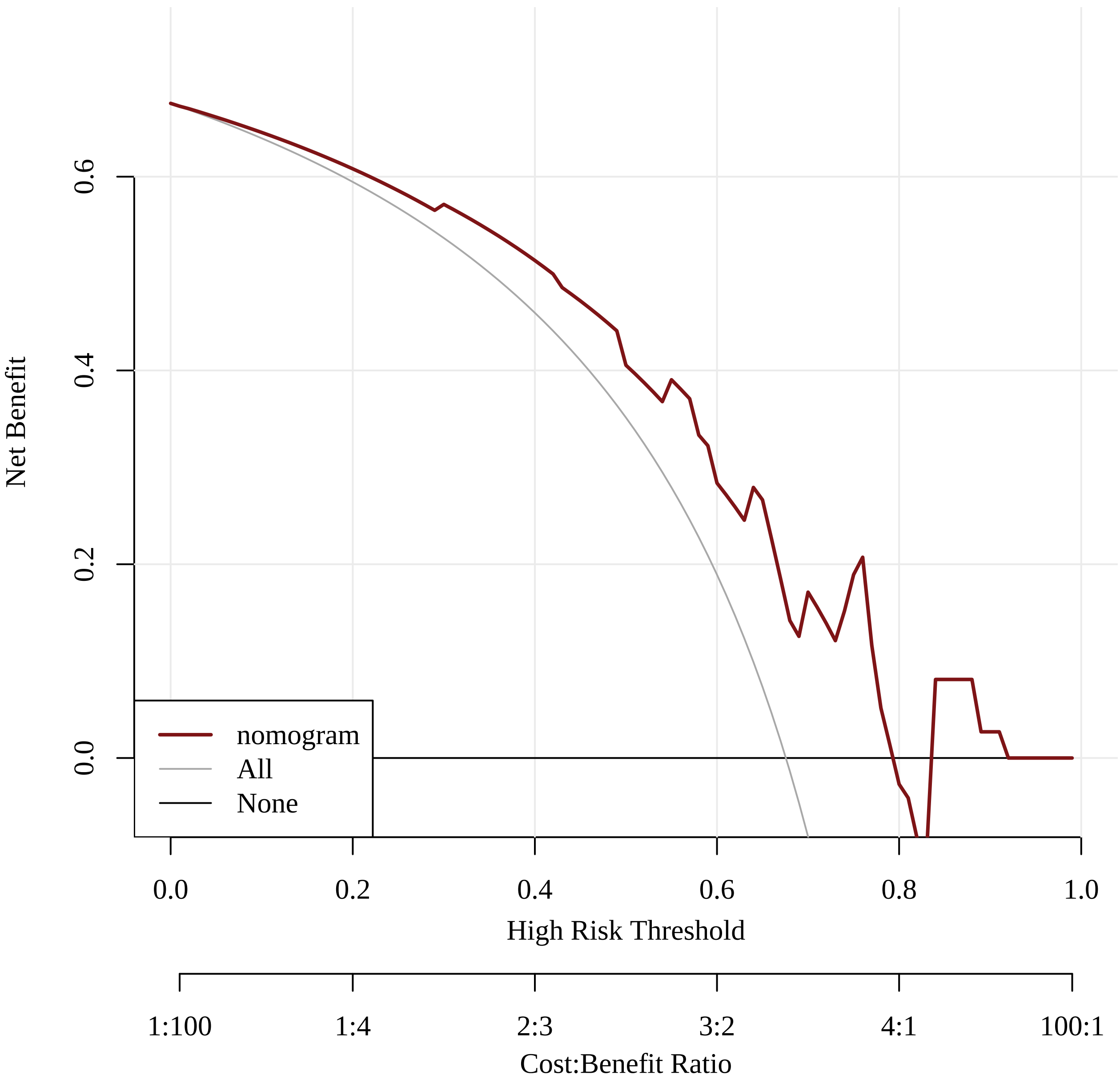

Net Benefit
0.0
0.2
0.4
0.6
nomogram
All
None
0.0
0.2
0.4
0.6
0.8
1.0
High Risk Threshold
1:100
1:4
2:3
3:2
4:1
100:1
Cost:Benefit Ratio
