# Supplementary material for: Identification of candidate selenium-associated transcriptomic biomarkers in chronic obstructive pulmonary disease: an integrated bioinformatics and expression validation study
Source: Front Med (Lausanne). 2026 Sep 30;13:1909444. doi: 10.3389/fmed.2026.1909444 (PMC13662921; doi:10.3389/fmed.2026.1909444)
Supplement: Supplementary file 1 [file Datasheet1.zip › Supplementary Materials/12_svm_sensitivity/04_auc_distribution.pdf]

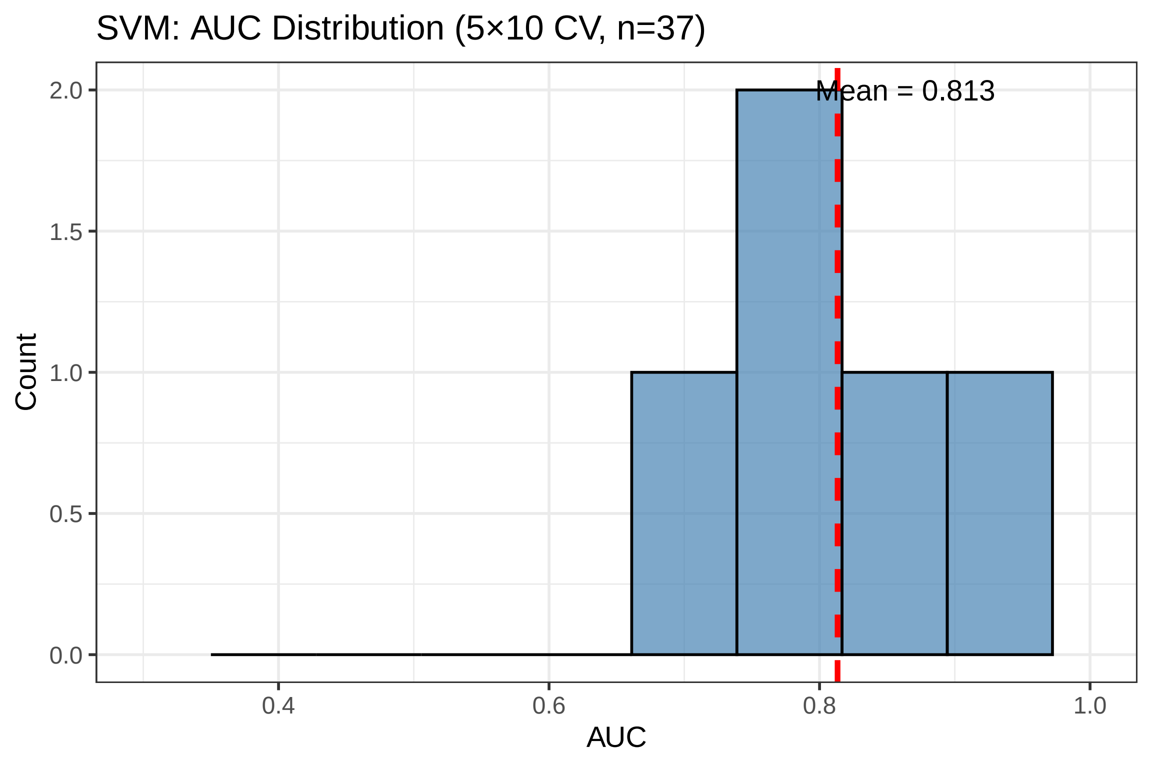
SVM: AUC Distribution (5×10 CV, n=37)
Mean = 0.813
Count
AUC
0.0
0.5
1.0
1.5
2.0
0.4
0.6
0.8
1.0
