# Supplementary material for: Identification of candidate selenium-associated transcriptomic biomarkers in chronic obstructive pulmonary disease: an integrated bioinformatics and expression validation study
Source: Front Med (Lausanne). 2026 Sep 30;13:1909444. doi: 10.3389/fmed.2026.1909444 (PMC13662921; doi:10.3389/fmed.2026.1909444)
Supplement: Supplementary file 1 [file Datasheet1.zip › Supplementary Materials/12_svm_sensitivity/05_gene_selection_frequency.pdf]

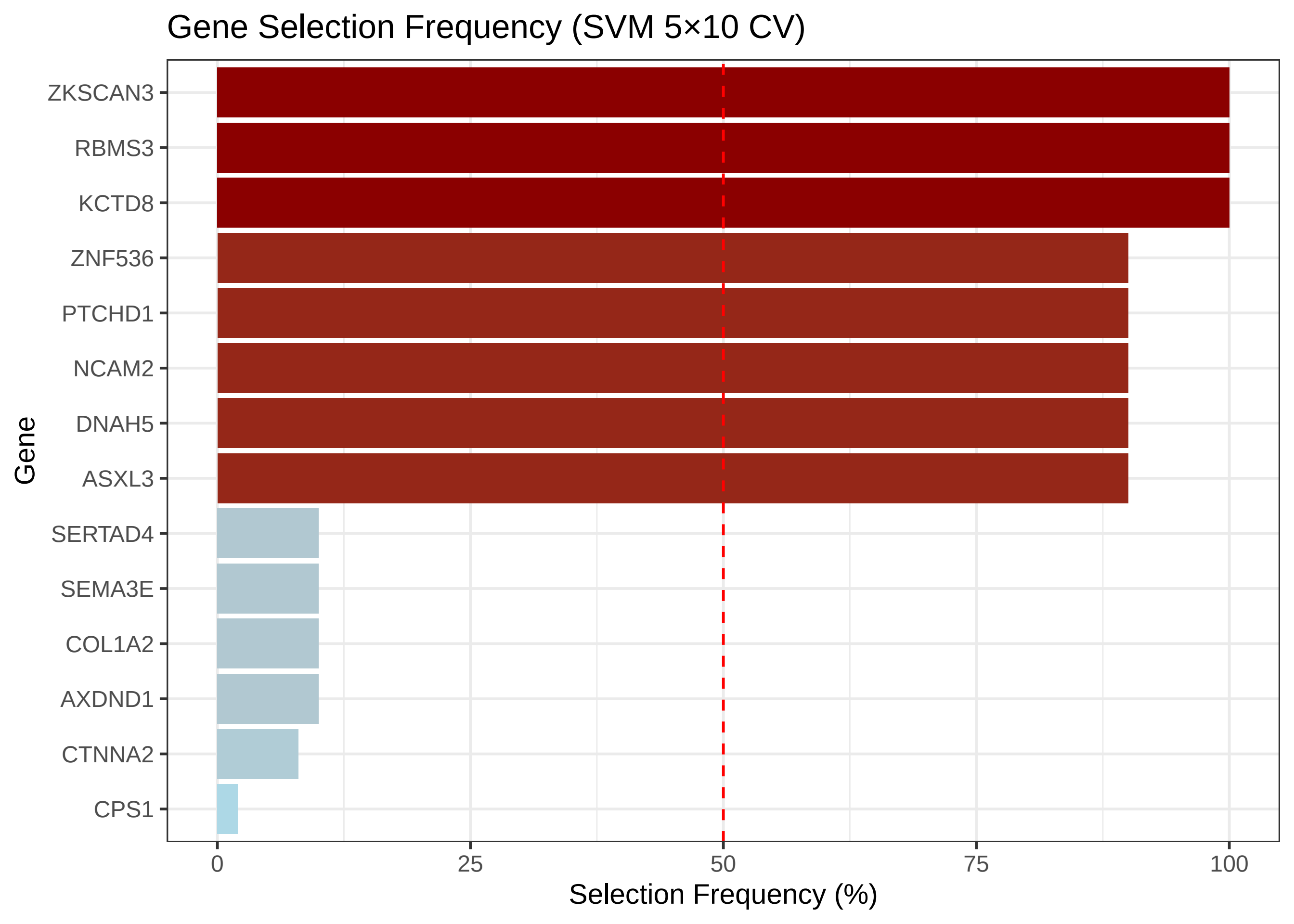
Gene Selection Frequency (SVM 5×10 CV)
ZKSCAN3
RBMS3
KCTD8
ZNF536
PTCHD1
NCAM2
DNAH5
ASXL3
SERTAD4
SEMA3E
COL1A2
AXDND1
CTNNA2
CPS1
Gene
0
25
50
75
100
Selection Frequency (%)
