# Supplementary material for: Identification of candidate selenium-associated transcriptomic biomarkers in chronic obstructive pulmonary disease: an integrated bioinformatics and expression validation study
Source: Front Med (Lausanne). 2026 Sep 30;13:1909444. doi: 10.3389/fmed.2026.1909444 (PMC13662921; doi:10.3389/fmed.2026.1909444)
Supplement: Supplementary file 1 [file Datasheet1.zip › Supplementary Materials/13_ssgsea/02.score_boxplot.pdf]

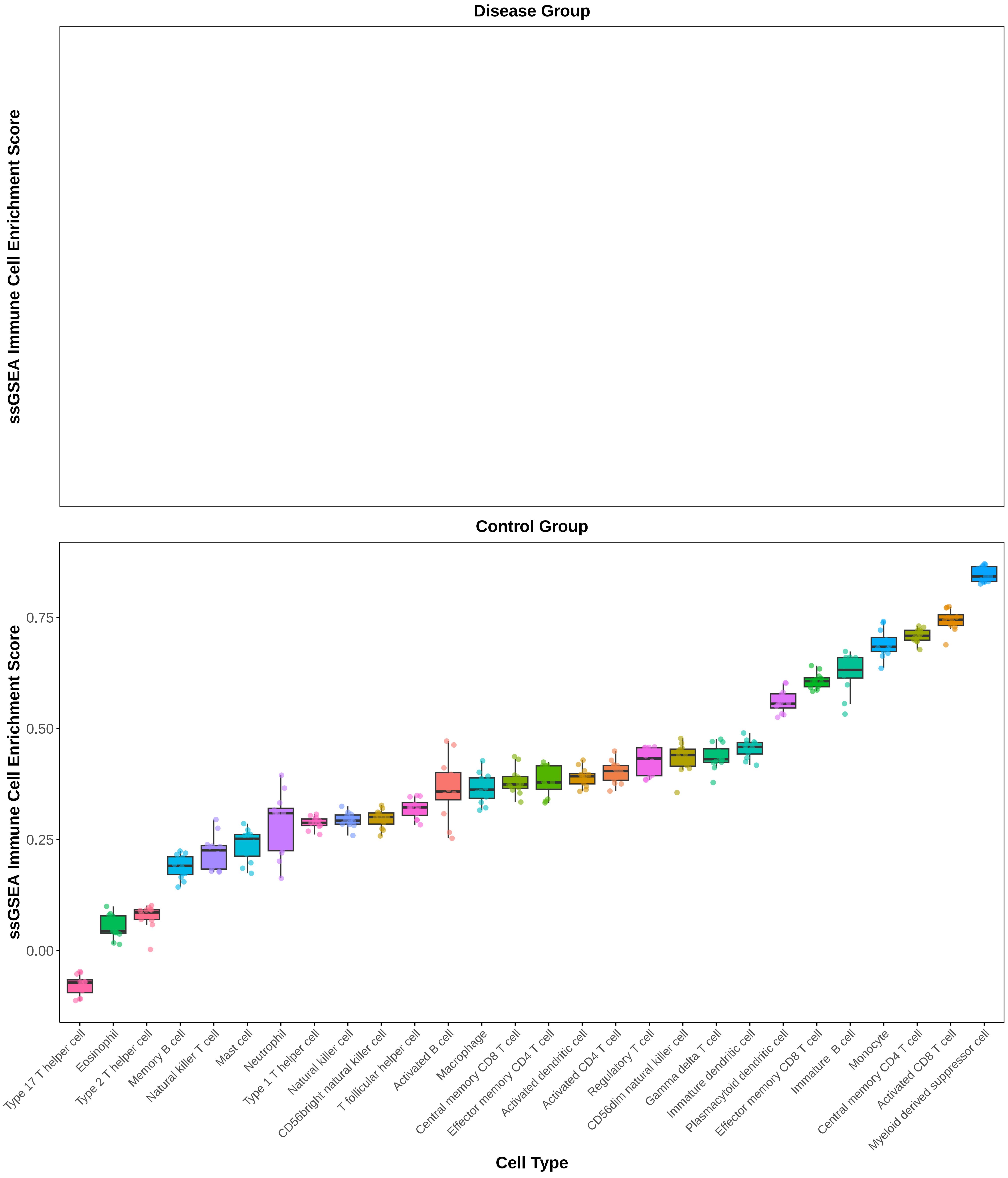
Disease Group
Control Group
ssGSEA Immune Cell Enrichment Score
ssGSEA Immune Cell Enrichment Score
0.75
0.50
0.25
0.00
Type 17 T helper cell
Eosinophil
Type 2 T helper cell
Memory B cell
Natural killer T cell
Mast cell
Neutrophil
Type 1 T helper cell
Natural killer cell
CD56bright natural killer cell
T follicular helper cell
Activated B cell
Macrophage
Central memory CD8 T cell
Effector memory CD4 T cell
Activated dendritic cell
Activated CD4 T cell
Regulatory T cell
CD56dim natural killer cell
Gamma delta T cell
Immature dendritic cell
Plasmacytoid dendritic cell
Effector memory CD8 T cell
Immature B cell
Monocyte
Central memory CD4 T cell
Activated CD8 T cell
Myeloid derived suppressor cell
Cell Type
