# Supplementary material for: Identification of candidate selenium-associated transcriptomic biomarkers in chronic obstructive pulmonary disease: an integrated bioinformatics and expression validation study
Source: Front Med (Lausanne). 2026 Sep 30;13:1909444. doi: 10.3389/fmed.2026.1909444 (PMC13662921; doi:10.3389/fmed.2026.1909444)
Supplement: Supplementary file 1 [file Datasheet1.zip › Supplementary Materials/13_ssgsea/04.ssGSEA_cell_boxplot.pdf]

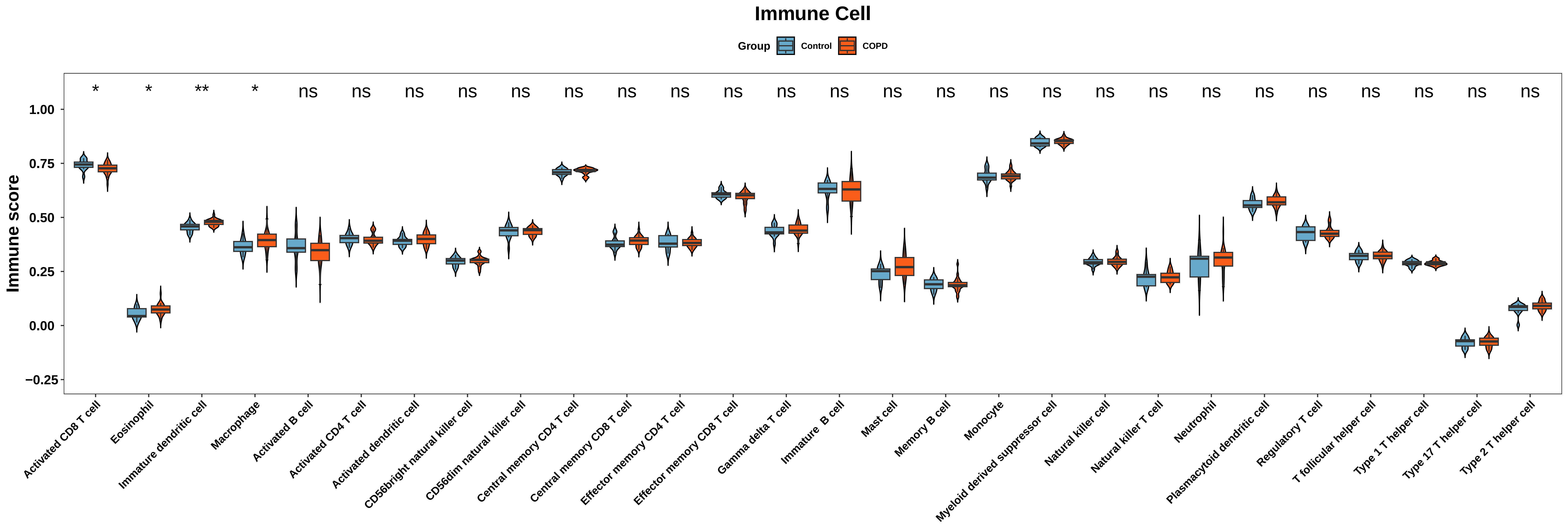
Immune Cell
Group
Control
COPD
Immune score
1.00
0.75
0.50
0.25
0.00
−0.25
*
*
**
*
ns
Activated CD8 T cell
Eosinophil
Immature dendritic cell
Macrophage
Activated B cell
Activated CD4 T cell
Activated dendritic cell
CD56bright natural killer cell
CD56dim natural killer cell
Central memory CD4 T cell
Central memory CD8 T cell
Effector memory CD4 T cell
Effector memory CD8 T cell
Gamma delta T cell
Immature B cell
Mast cell
Memory B cell
Monocyte
Myeloid derived suppressor cell
Natural killer cell
Natural killer T cell
Neutrophil
Plasmacytoid dendritic cell
Regulatory T cell
T follicular helper cell
Type 1 T helper cell
Type 17 T helper cell
Type 2 T helper cell
