# Supplementary material for: Identification of candidate selenium-associated transcriptomic biomarkers in chronic obstructive pulmonary disease: an integrated bioinformatics and expression validation study
Source: Front Med (Lausanne). 2026 Sep 30;13:1909444. doi: 10.3389/fmed.2026.1909444 (PMC13662921; doi:10.3389/fmed.2026.1909444)
Supplement: Supplementary file 1 [file Datasheet1.zip › Supplementary Materials/13_ssgsea/07.Correlation_cell_genes.pdf]

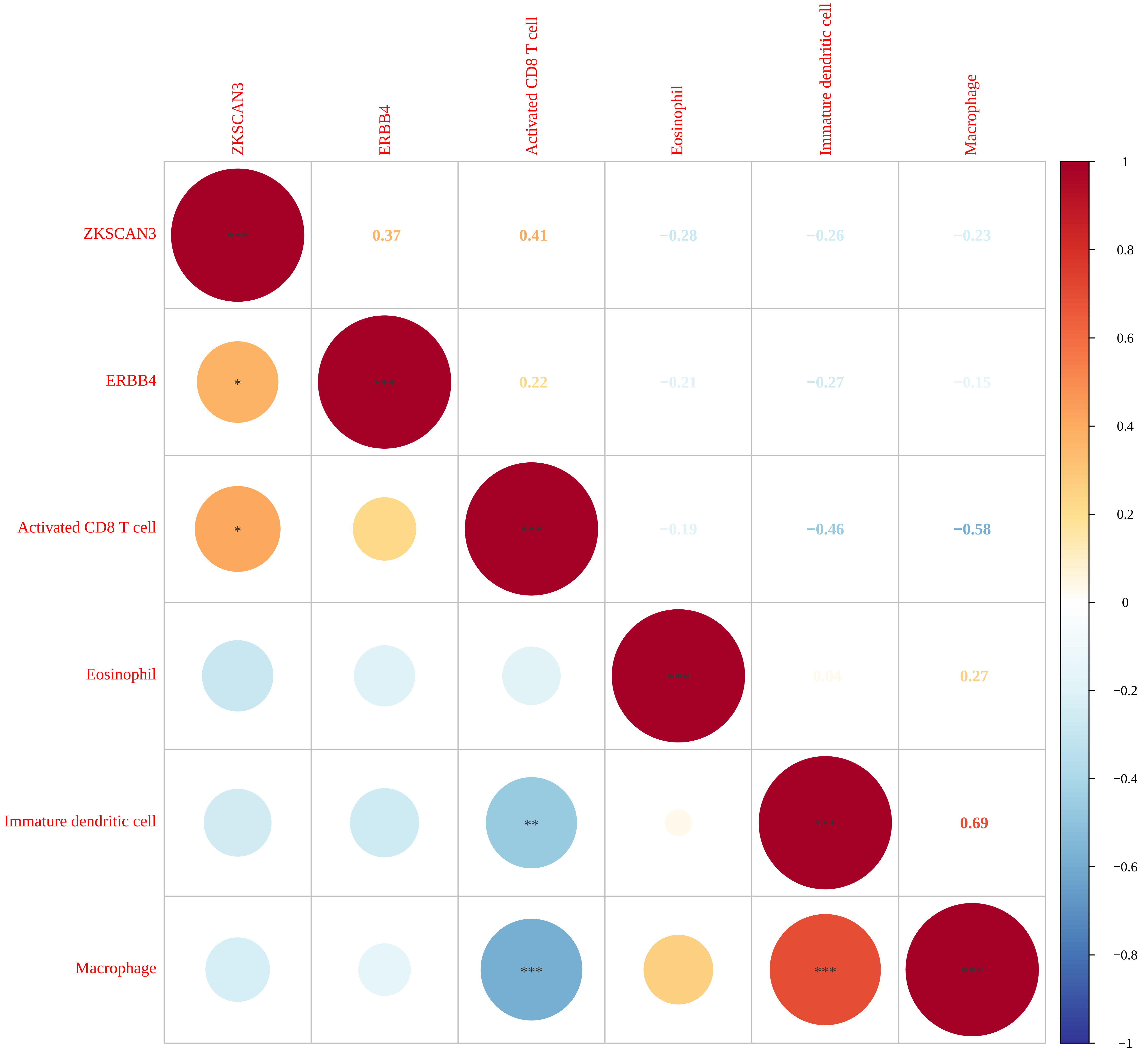

ZKSCAN3
ERBB4
Activated CD8 T cell
Eosinophil
Immature dendritic cell
Macrophage
0.37
0.41
−0.28
−0.26
−0.23
0.22
−0.21
−0.27
−0.15
−0.19
−0.46
−0.58
0.04
0.27
0.69
*
*
**
***
1
0.8
0.6
0.4
0.2
0
−0.2
−0.4
−0.6
−0.8
−1
