# Supplementary material for: Identification of candidate selenium-associated transcriptomic biomarkers in chronic obstructive pulmonary disease: an integrated bioinformatics and expression validation study
Source: Front Med (Lausanne). 2026 Sep 30;13:1909444. doi: 10.3389/fmed.2026.1909444 (PMC13662921; doi:10.3389/fmed.2026.1909444)
Supplement: Supplementary file 1 [file Datasheet1.zip › Supplementary Materials/Supplementary_Material.docx]

Supplementary Material

# 1 Supplementary Tables

For more information on Supplementary Material and for details on the different file types accepted, please see [here](https://www.frontiersin.org/guidelines/author-guidelines#supplementary-material).

## 1.1 Supplementary Tables

**Supplementary Table 1. Selenium metabolism and selenoprotein genes collected from the WikiPathways term “WP_SELENIUM_METABOLISM_AND_SELENOPROTEINS” in the C2 curated canonical pathway subset of MSigDB.**

**Supplementary Table 2. Primer sequences used for RT-qPCR validation of ERBB4 and ZKSCAN3 expression.**

**Supplementary Table 3. Differentially expressed genes identified between COPD and control samples in GSE248493.**

**Supplementary Table 4. Selenium metabolism and selenoprotein-related key genes identified from the WGCNA key module.**

**Supplementary Table 5. Gene Ontology enrichment results of the 17 candidate genes, including biological process, cellular component, and molecular function terms.**

**Supplementary Table 6. Kyoto Encyclopedia of Genes and Genomes pathway enrichment results of the 17 candidate genes.**

**Supplementary Table 7. Gene set enrichment analysis results for ERBB4 and ZKSCAN3 in GSE248493.**

**Supplementary Table 8. COPD Clinical Information Sheet**
